# ﻿Global diversity of the *Tylopilus alboater* complex (*Boletaceae*, *Boletales*): new genus and species, and typification of the name *Boletus alboater*

**DOI:** 10.3897/imafungus.16.159676

**Published:** 2025-10-31

**Authors:** Jin Li, Roy E. Halling, Todd W. Osmundson, Zhu L. Yang, Yan-Chun Li

**Affiliations:** 1 Key Laboratory of Phytochemistry and Natural Medicines, Kunming Institute of Botany, Chinese Academy of Sciences, Lanhei Road, Kunming, 650201, China Chinese Academy of Sciences Kunming China; 2 Yunnan Key Laboratory for Fungal Diversity and Green Development, Lanhei Road, Kunming, 650201, China Yunnan Key Laboratory for Fungal Diversity and Green Development Kunming China; 3 University of Chinese Academy of Sciences, Yanqi Lake East Road, Beijing 100049, China University of Chinese Academy of Sciences Beijing China; 4 Center for Diversity & Evolution, The New York Botanical Garden, Bronx, NY 10458-5126, USA The New York Botanical Garden New York United States of America; 5 Department of Biology, University of Wisconsin–La Crosse, 1725 State Street, La Crosse, WI 54601, USA University of Wisconsin–La Crosse La Crosse United States of America

**Keywords:** *Boletes*, morphology, new taxa, phylogeny, taxonomy

## Abstract

Recognition of *Tylopilus alboater* once relied heavily on the morphological features of the basidiomata, which resulted in considerable confusion due to morphological stasis and plasticity. In this study, we examined specimens morphologically identified as *T. alboater* from Asia, North America, and Australia using phylogenomics and multi-locus sequence data. To clarify its phylogenetic placement within *Boletaceae*, we conducted a phylogenomic analysis based on 45 genomes (including three newly sequenced *T. alboater* genomes) comprising representatives from the eight subfamilies of *Boletaceae*. Additionally, we constructed a concatenated dataset (nrLSU + *tef1-α* + *rpb1* + *rpb2*), incorporating representative species from all genera within the subfamily to which *T. alboater* belongs, to infer its phylogeny. Our phylogenomic analysis revealed that specimens morphologically identified as *T. alboater* exhibit polyphyly, clustering entirely within the subfamily *Boletoideae*. Our multi-locus phylogenetic analyses further indicated that these specimens represent eight distinct species distributed across five generic lineages within the subfamily *Boletoideae*, including one new genus, *Neoporphyrellus*, proposed in this study, as well as one new species, *N. sinoalboater*, and two new combinations, *N. alboater* and *N. atronicotianus*. The remaining five species are nested in four known genera, including two new species of *Abtylopilus*, *Ab. indonesiensis* and *Ab. australiensis*, and three known species belonging to *Anthracoporus*, *Indoporus*, and *Tylopilus*, respectively. These findings highlight the taxonomic complexity of the *T. alboater* complex and emphasize the importance of integrating phylogenomic and multi-locus approaches for accurate fungal systematics. Color photos of fresh basidiomata, line drawings of microscopic features, and detailed descriptions of the new taxa are presented.

## ﻿Introduction

The genus *Tylopilus* P. Karst. was proposed by Karsten (1881) and typified by *T. felleus* (Bull.) P. Karst. Most species traditionally attributed to this genus have white, pinkish, purplish pink, reddish, reddish brown, or brownish hymenophores with pink to pinkish brown spore prints. With the development and utilization of molecular techniques, phylogenetic analyses based on multigene sequences indicated that the original concepts of *Tylopilus* were polyphyletic, and many new genera were proposed based on species formerly placed in this genus, viz. *Zangia* Yan C. Li & Zhu L. Yang, *Harrya* Halling, Nuhn & Osmundson, *Sutorius* Halling, Nuhn & N.A. Fechner, *Pseudoaustroboletus* Yan C. Li & Zhu L. Yang, *Chiua* Yan C. Li & Zhu L. Yang, *Hymenoboletus* Yan C. Li & Zhu L. Yang, *Tylocinum* Yan C. Li & Zhu L. Yang, *Indoporus* A. Parihar, K. Das, Hembrom & Vizzini, *Anthracoporus* Yan C. Li & Zhu L. Yang, *Abtylopilus* Yan C. Li & Zhu L. Yang, *Brasilioporus* A.C. Magnago, Alves-Silva & T.W. Henkel, *Nevesoporus* A.C. Magnago & T.W. Henkel, and *Kgaria* Halling, Fechner & Davoodian (Li et al. 2011, 2014; Halling et al. 2012a, 2012b; Wu et al. 2016a; Parihar et al. 2018; Li and Yang 2021; Magnago et al. 2022; Halling et al. 2023). Among these genera, six of them—viz., *Abtylopilus*, *Anthracoporus*, *Brasilioporus*, *Nevesoporus*, *Kgaria*, and *Indoporus*—have common morphological characteristics: dark-colored basidiomata and initially reddish, then blackish, discoloration of the context when injured. Such traits are also shared by *Tylopilus alboater* (Schwein.) Murrill. *Tylopilus alboater* was originally described as *Boletus alboater* Schwein. from North America by Schweinitz (1822). Kuntze (1898) transferred it to *Suillus* Gray, and Gilbert (1931) then transferred it to *Porphyrellus* based on its umber basidiomata. Murrill (1909) transferred it to *Tylopilus* based on its dull pink or flesh-colored hymenophore and rosy to flesh-colored basidiospores, a treatment followed by Singer (1947) and Smith and Thiers (1971). Due to morphological stasis and plasticity, *T. alboater* was regarded as a single polymorphic species distributed in Asia, North America, and Australia (GBIF: https://www.gbif.org/, data accessed 10 April 2025). However, whether the samples identified as *T. alboater* from such a vast geographic scope represent a single species has not been specifically addressed.

In this study, eleven samples identified as *T. alboater* from Asia, Australia, and North America were analyzed based on molecular phylogenetic analyses using nuclear genes: the nuclear ribosomal large subunit (nrLSU), the translation elongation factor 1-α gene (*tef1-α*), the largest subunit of RNA polymerase II (*rpb1*), and the second-largest subunit of RNA polymerase II (*rpb2*), along with morphological and ecological data. A new genus, *Neoporphyrellus*, including one new species and two new combinations, and two new species belonging to *Abtylopilus* are proposed. The aims of this paper are to (1) investigate the phylogenetic position of species identified as *T. alboater* from Australia, East Asia, and North America; (2) evaluate the relationships among species in *Neoporphyrellus*; and (3) compare the morphological features of the new genus *Neoporphyrellus* with similar genera *Abtylopilus*, *Anthracoporus*, *Brasilioporus*, *Nevesoporus*, and *Indoporus*.

## ﻿Materials and methods

### ﻿Morphological studies

Macromorphological characteristics were derived from sample records and photographs of the fresh basidiomata. Color codes used for the newly described taxa are based on Kornerup and Wanscher (1981). Microscopic characteristics were examined under light microscopy after sectioning and mounting tissues in 5%–10% KOH solution. The features assessed included the structure of the pileipellis, the morphology of the basidia and cystidia, and the morphology and ornamentation of the basidiospores. The amyloidity and dextrinoidity of the basidiospores were tested in Melzer’s reagent. All microscopic structures illustrated here were drawn freehand from rehydrated materials. In the descriptions of basidiospores, the abbreviation n/m/p means *n* basidiospores measured from *m* basidiomata of *p* samples. The notation of the form (a) b–c (d) stands for the dimensions of the basidiospores; the range b–c contains 90% of the measured values, and *a* or *d*, given in parentheses, represents the extreme values. *Q* refers to the length/width ratio of a basidiospore in profile view, and *Qm* is the average *Q* of all basidiospores ± sample standard deviation. Voucher specimens from our field investigations are deposited in the fungal herbarium of the Herbarium KUN and compared with specimens in NY, BPI, FH, K, PH, and UPS. Herbarium codes follow Thiers (2025).

### ﻿Genome sequencing, assembly, ortholog extraction, and phylogenomic analysis

To clarify the phylogenetic placement of “*Tylopilus alboater*” within *Boletaceae*, a phylogenomic analysis was conducted. Three samples identified as *Tylopilus alboater* were newly sequenced on an Illumina HiSeq platform, following the protocols described in Zeng et al. (2018). Raw data quality was controlled using Fastp v0.23.4 (Chen et al. 2018). Genome assembly was performed with SPAdes v3.15.5 (Bankevich et al. 2012) using automatic *k*-mer selection based on reads length.

According to the phylogenomic study of Tremble et al. (2024), we selected 45 genomes representing taxa of the eight subfamilies of *Boletaceae*, combined with the three newly sequenced genomes of *T. alboater*, for phylogenomic analysis. Two genomes of *Suillus (Suillaceae)* and two genomes of *Paxillus (Paxillaceae)* were selected as outgroup taxa. Assembly completeness and ortholog extraction of all genomes were performed using BUSCO v5.8.2 (Manni et al. 2021a, b) with the “basidiomycota_odb12” dataset, following the extraction protocols of Tremble et al. (2024) and Wang et al. (2024). Selected orthologs were aligned using MAFFT v7.525 (Katoh et al. 2019) with the L-INS-i strategy, and conserved sequences were extracted using Gblocks v0.91b (Castresana 2000) with default parameters. Phylogenomic maximum-likelihood gene trees were inferred using IQ-TREE v2.4.0 (Minh et al. 2020) with ultrafast bootstrapping (Hoang et al. 2018) of 1,000 replicates. The model for each gene was selected automatically in ModelFinder (Kalyaanamoorthy et al. 2017). Sample information is provided in Suppl. material 1.

### ﻿Molecular procedures and phylogenetic analyses

Total DNA was extracted from silica gel–dried or herbarium materials using the CTAB method (Doyle and Doyle 1987). PCR, sequencing, and sequence alignment followed those described in Liu et al. (2021), Orihara et al. (2021), Mao et al. (2023), Shen et al. (2023), Tang et al. (2023), and references therein. The phylogenetic analyses were based on four nuclear loci: nrLSU, *tef1-α*, *rpb1*, and *rpb2*. The common primer pairs LROR/LR5, 983F/1567R, rpb1-Af/rpb1-Cr, and rpb2-6F/rpb2-7R were used to amplify the above gene fragments, respectively. DNA sequences were compiled with SeqMan (DNASTAR Lasergene 9). Alignments were constructed separately for each of the gene fragments using MAFFT v7.310 (Katoh and Standley 2013) with the L-INS-i strategy. Suboptimal gap placements, homology displacement, chimeric artifacts, or sequencing-induced mutations were manually corrected. The alignments were then concatenated using PhyloSuite v1.2.3 (Zhang et al. 2020), with unsampled gene regions coded as missing data. Sequences newly generated in this study have been submitted to GenBank. Detailed information on voucher specimens, including GenBank accession numbers, is provided in Table 1. Genera are abbreviated as follows: *Ab.* for *Abtylopilus*, *An.* for *Anthracoporus*, *Af.* for *Afroboletus* Pegler & T.W.K. Young, *Af.* for *Afrocastellanoa* M.E. Smith & Orihara, *B.* for *Boletus*, *Br.* for *Brasilioporus*, *Bu.* for *Butyriboletus* D. Arora & J.L. Frank, *E.* for *Erythrophylloporus* Ming Zhang & T.H. Li, *G.* for *Guyanaporus* T.W. Henkel & M.E. Sm., *H.* for *Hortiboletus* Simonini, Vizzini & Gelardi, *I.* for *Indoporus*, *Im.* for *Imleria* Vizzini, *J.* for *Jimtrappea* T.W. Henkel, M.E. Smith & Aime, *K.* for *Kgaria*, *N.* for *Neoporphyrellus*, *N..* for *Neotropicomus* A.C. Magnago, Alves-Silva & T.W. Henkel, *N.v.* for *Nevesoporus* A.C. Magnago & T.W. Henkel, *P.* for *Porphyrellus* E.-J. Gilbert, *Pa.* for *Parvixerocomus* G. Wu & Zhu L. Yang, *Pax.* for *Paxilloboletus* Furneaux, De Kesel & F.K. Khan, *S.* for *Strobilomyces* Berk., *T.* for *Tylopilus*, *T..* for *Tengioboletus* G. Wu & Zhu L. Yang, and *X.* for *Xanthoconium* Singer.

**Table 1.** Information on the taxa used in the phylogenetic analyses. Names, voucher numbers, localities, and their corresponding GenBank accession numbers are listed here. Sequences obtained in this study are shown in bold. “–” represents missing data.

Species	Voucher	Locality	Accession				References
			nrLSU	rpb1	rpb2	tef1-α	
*Abtylopilus alborubellus*	HKAS 99704 type	China	MT154713	–	–	–	Li and Yang 2021
*Ab. australiensis*	NY 2049854	Australia	**OR122506 OR122507**	**OR130499 OR130500**	**OR130501 OR130502**	**OR130503 OR130504**	**This study**
*Ab. indonesiensis*	NY 1393740	Indonesia	**OQ690002**	–	**OQ689705**	**OQ689702**	**This study**
*Ab. scabrosus*	HKAS 59826	China	KT990558	MT110379	–	MT110336	Li and Yang 2021
*Ab. scabrosus*	HKAS 50211 type	China	KT990552	KT990920	KT990389	KT990752	Li and Yang 2021
*Afroboletus luteolus*	Yorou 3141	Zambia	–	KY553114	KY553124	KY553134	Han et al. 2017
*Af. luteolus*	Yorou 4599	Madagascar	–	KY553115	KY553125	KY553135	Han et al. 2017
*Af. multijugus*	PC 0723572	Burundi	–	KY553118	KY553128	KY553138	Han et al. 2017
*Af. multijugus*	PC 0723578	Madagascar	–	KY553117	KY553127	KY553137	Han et al. 2017
*Afrocastellanoa ivoryana*	OSC 150014 type	Zimbabwe	KX685720	KX685715	–	–	Oriharaa and Smith 2017
*Anthracoporus cystidiatus*	HKAS 55375	China	KT990622	KT990969	MT110410	KT990816	Li and Yang 2021
*An. cystidiatus*	NY 2049837	Thailand	**OQ690003**	–	–	–	**This study**
*An. cystidiatus*	NY 2072261	Thailand	**OQ690001**	–	**PV189435**	**OQ689700**	**This study**
*An. cystidiatus*	MHHNU 7312 type	China	MT154710	MT110377	MT110411	–	Li and Yang 2021
*An. holophaeus*	HKAS 59407	China	KT990708	KT991030	KT990506	KT990888	Wu et al. 2016a
*An. holophaeus*	HKAS 50508	China	–	KX869634	KX869506	KX869376	Han et al. 2018
*An. nigropurpureus*	HKAS 52685	China	KT990627	KT990973	–	KT990821	Wu et al. 2016a
*An. nigropurpureus*	HKAS 53370	China	KT990628	KT990974	KT990460	KT990822	Wu et al. 2016a
*Boletus edulis*	HMJAU 4637	China	KF112455	KF112586	KF112704	KF112202	Wu et al. 2014
*B. reticuloceps*	HKAS 51232	China	KT990537	KT990906	KT990376	KT990739	Wu et al. 2016a
*B. reticuloceps*	HKAS 57671	China	KF112454	KF112648	KF112703	KF112201	Wu et al. 2014
*Brasilioporus olivaceoflavidus*	VIES 9901322 type	Brazil	NG088318	OM160565	OM160576	OM160555	Magnago et al. 2022
*Br. olivaceoflavidus*	VIES 9901323	Brazil	OM068913	OM160566	–	OM160556	Magnago et al. 2022
*Br. rufonigricans*	TH 6376	Guyana	AY612835	–	–	–	Magnago et al. 2022
*Br. simoniarum*	VIES 9901327 type	Brazil	NG088319	OM160567	OM160577	OM160557	Magnago et al. 2022
*Butyriboletus roseoflavus*	HKAS 53405	China	KF739666	KF739780	KF739742	KF739704	Wu et al. 2016a
*Bu. roseoflavus*	HKAS 54099	China	KF739665	KF739779	KF739741	KF739703	Wu et al. 2016a
*Erythrophylloporus cinnabarinus*	GDGM 44440	China	MH374032	MH378801	MH374029	MH374033	Zhang and Li 2018
*E. cinnabarinus*	GDGM 70536 type	China	MH374045	MH378802	MH374031	MH374035	Zhang and Li 2018
*Guyanaporus albipodus*	Henkel 8848 type	Guyana	HQ161868	–	LC043082	LC043083	Henkel et al. 2016
*Hortiboletus amygdalinus*	HKAS 54242	China	KT990580	–	KT990415	KT990776	Wu et al. 2016a
*H. amygdalinus*	HKAS 54166 type	China	KT990581	KT990933	KT990416	KT990777	Wu et al. 2016a
*H. subpaludosus*	HKAS 68158	China	KT990583	KT990934	KT990418	KT990779	Wu et al. 2016a
*H. subpaludosus*	HKAS 59608	China	KF112371	KF112551	KF112696	KF112185	Wu et al. 2014
*Indoporus shoreae*	AP 6693 type	India	MK123973	–	MK243367	–	Parihar et al. 2018
*I. shoreae*	AP 6697	India	MK123976	–	MK243368	–	Parihar et al. 2018
*I. squamulosus*	HKAS 107153	China	MT154708	MT110375	–	MT110334	Li and Yang 2021
*I. squamulosus*	HKAS 76299 type	China	MT154709	MT110376	MT110409	MT110335	Li and Yang 2021
*I. squamulosus*	NY 2049839	Thailand	**OQ690004**	–	**OQ689704**	**OQ689701**	**This study**
*Jimtrappea guyanensis*	Henkel 9163	Guyana	LC053660	–	LC053661	–	Smith et al. 2015
*Kgaria cyanogranulifer*	NY 1194066	Australia	JX889646	JX889688	–	OR263680	Halling et al. 2023
*K. cyanogranulifer*	NY 1194065	Australia	JX889647	JX889689	–	OR263681	Halling et al. 2023
*K. similis*	NY 1193839	Australia	OR063867	OR113660	–	OR263685	Halling et al. 2023
*K. similis*	NY 1193840	Australia	OR063869	–	–	OR263686	Halling et al. 2023
*Imleria badia*	Xb2	Germany	KF030357	–	–	KF030422	Nuhn et al. 2013
*Im. badia*	HKAS 74714	China	KF112375	KF112609	–	–	Wu et al. 2014
*Im. parva*	HKAS 55341 type	China	KC215216	KC215229	KC215238	KC215252	Zhu et al. 2014
*Im. parva*	HKAS 59437	China	KC215215	KC215228	KC215237	KC215250	Zhu et al. 2014
*Neoporphyrellus alboater*	TH 6941	USA	AY612832	–	–	–	Drehmel et al. 2008
*N. alboater*	NY 1193926	USA	**OP771514**	**OP765296**	**OP762644**	**OP750052**	**This study**
*N. atronicotianus*	snWV	USA	KF030293	–	–	–	Nuhn et al. 2013
*N. atronicotianus*	CFMR 160.	USA	EU685110	–	–	–	Desjardin et al. 2009
*N. atronicotianus*	NY 815170 paratype	USA	**OQ642297**	–	–	**PV189431**	**This study**
*N. atronicotianus*	NY 815171 paratype	USA	**OQ642298**	–	**OQ689703**	**OQ689699**	**This study**
*N. sinoalboater*	HKAS 107186	China	**OP771513**	**OP765295**	**OP762643**	**PV189432**	**This study**
*N. sinoalboater*	HKAS 78815	China	**OP771512**	**PV189433**	**PV189438**	**OP750051**	**This study**
*N. sinoalboater*	HKAS 145312	China	**PQ725682**	–	**PV189436**	**PV189429**	**This study**
*N. sinoalboater*	HKAS 145313	China	**PQ725682**	**PV189434**	**PV189437**	**PV189430**	**This study**
*Neotropicomus australis*	VIES 9901329	Brazil	OM068917	–	–	–	Magnago et al. 2022
*N.. australis*	ICN 202156	Brazil	OM068916	OM160572	–	–	Magnago et al. 2022
*N.. parvogracilis*	TH 9209 type	Guyana	NG059946	–	–	–	Magnago et al. 2022
*Nevesoporus exiguus*	TH 9549	Guyana	KT339205	–	–	–	Magnago et al. 2022
*N.v. nigrostipitatus*	VIES 9901383 type	Brazil	NG149037	–	–	OM160562	Magnago et al. 2022
*N.v. nigrostipitatus*	VIES 9901384	Brazil	OM068919	–	–	–	Magnago et al. 2022
*Parvixerocomus aokii*	HKAS 59812	China	KF112378	–	–	KF112266	Wu et al. 2014
*Pa. pseudoaokii*	HKAS 52633	China	KF112379	KF112598	KF112736	KF112267	Wu et al. 2014
*Pa. pseudoaokii*	HKAS 80480 type	China	KP658468	KP658472	KP658470	–	Wu et al. 2016b
*Pa. pseudoaokii*	HKAS 77032	China	KP658467	KP658471	KP658469	–	Wu et al. 2016b
*Paxilloboletus africanus*	SAB 0716 type	Guinea	MZ702479	MZ707865	MZ707878	MZ707869	Badou et al. 2022
*Pax. latisporus*	ADK 5072 type	DR Congo	MZ702481	MZ707866	MZ707879	MZ707870	Badou et al. 2022
*Porphyrellus cyaneotinctus*	HKAS 80183	China	MT154718	–	–	MT110340	Li and Yang 2021
*Po. cyaneotinctus*	HKAS 80192	China	MT154719	–	–	–	Li and Yang 2021
*Po. porphyrosporus*	HKAS 48585	China	KT990543	KT990911	KT990382	KT990745	Wu et al. 2016a
*Po. porphyrosporus*	HKAS 49182	China	KT990544	KT990912	KT990383	KT990746	Wu et al. 2016a
*Strobilomyces seminudus*	HKAS 82848	China	–	KT990985	KT990472	KT990835	Wu et al. 2016a
*S. seminudus*	HKAS 80400	China	–	APA20757	APA20446	APA20607	Wu et al. 2016a
*S. verruculosus*	HKAS 59637	China	–	–	APA20450	APA20611	Wu et al. 2016a
*S. verruculosus*	HKAS 77026	China	–	APA20761	APA20450	APA20611	Wu et al. 2016a
*Tylopilus felleus*	HKAS 54926	China	KF112411	KF112575	KF112737	HQ326866	Wu et al. 2014
*T. himalayanus*	DC 17-25	India	MG799328	–	–	–	Chakraborty et al. 2018
*T. himalayanus*	HKAS 91278	China	MT154742	MT110392	MT110427	MT110354	Li and Yang 2021
*T. jiangxiensis*	HKAS 107152 type	China	MT154731	–	–	–	Li and Yang 2021
*T. jiangxiensis*	HKAS 105250	China	MN304779	MN304785	MN304791	MN304797	Zhao et al. 2020
*T. rubrobrunneus*	HKAS 19069	USA	**OQ819424**	**OQ828460**	**OQ828460**	**OQ828458**	**This study**
*T. rubrobrunneus*	BD 329	USA	HQ161876	–	HQ161845	–	Dentinger et al. 2010
*Tengioboletus glutinosus*	HKAS 53425 type	China	KF112341	KF112578	KF112800	KF112204	Wu et al. 2014
*T.. glutinosus*	HKAS 53452	China	KT990655	KT990994	KT990480	KT990844	Wu et al. 2016a
*T.. reticulatus*	HKAS 53426	China	KF112491	KF112649	KF112828	KF112313	Wu et al. 2014
*T.. reticulatus*	HKAS 53453 type	China	KT990656	–	KT990482	KT990846	Wu et al. 2016a
*Xanthoconium purpureum*	BD 228	USA	HQ161864	HQ16183	–	–	Dentinger et al. 2010
*X. purpureum*	NY 00720964	USA	KT990663	KT991001	–	KT990852	Wu et al. 2016a
*X. sinense*	HKAS 80118	China	KT990666	KT991004	KT990490	KT990855	Wu et al. 2016a
*X. sinense*	HKAS 77758 type	China	KT990665	KT991003	KT990489	KT990854	Wu et al. 2016a
*Xerocomellus chrysenteron*	HKAS 56494	China	KF112357	KF112526	KF112685	KF112172	Wu et al. 2014
*Xe. corneri*	HKAS 90206	China	KT990669	KT991007	KT990493	KT990857	Wu et al. 2016a
*Xe. corneri*	HKAS 52503	China	KT990668	KT991006	KT990492	KT990856	Wu et al. 2016a
*Xe. roseonigrescens*	GDGM 43238 type	China	KT220588	KT220591	KT220593	KT220595	Gelardi et al. 2015
*Xe. roseonigrescens*	ZT 13553	China	KT220589	KT220592	KT220594	KT220596	Gelardi et al. 2015

Forty-six sequences (14 for nrLSU, 13 for *tef1-α*, 7 for *rpb1*, and 12 for *rpb2*) from thirteen samples were newly generated in this study and aligned with selected sequences from GenBank and previous studies (Desjardin et al. 2009; Dentinger et al. 2010; Nuhn et al. 2013; Wu et al. 2014, 2016a, 2016b; Chakraborty et al. 2018; Han et al. 2017, 2018; Parihar et al. 2018; Li and Yang 2021; Magnago et al. 2022) (Table 1). *Butyriboletus roseoflavus* (Hai B. Li & Hai L. Wei) D. Arora & J.L. Frank and *E. cinnabarinus* Ming Zhang & T.H. Li were chosen as outgroup taxa (Wu et al. 2016; Zhang and Li 2018). For protein-coding genes, *tef1-α* was divided into five blocks (three exons and two introns), *rpb1* into four blocks (two exons and two introns), and *rpb2* into three blocks (two exons and one intron). The nrLSU was treated as a single block. Thus, the dataset was partitioned into 13 partitions. The combined nuclear dataset was analyzed using Maximum Likelihood (ML) and Bayesian Inference (BI). The Approximately Unbiased (AU) test was performed with IQ-TREE v2.2.6 using parameters “-m MFP+MERGE -bb 1000 -zb 10000” to evaluate topological incongruence among the four genes (Shimodaira 2000; Minh et al. 2020). Conflicts were observed among gene trees (Suppl. material 2), manifested as topological conflicts in individual loci. Nevertheless, concatenated analysis was conducted because multi-locus data integration can statistically compensate for single-gene discordance, enhance reconstruction of species divergence history, and resolve phylogenetic relationships with much stronger nodal support (Xi et al. 2013; Wascher and Kubatko 2021). The model for each partition was selected independently. The ML analysis was performed with IQ-TREE v2.2.6, with automatic model selection in ModelFinder for each partition (Kalyaanamoorthy et al. 2017) and ultrafast bootstrapping (Hoang et al. 2018) with 1,000 replicates. The best model for each partition is provided in Suppl. material 3. The BI analysis was performed with MrBayes v3.2.7a, running 5 million bootstrap replicates combined with a BI search (Ronquist et al. 2012). The parameter model was selected by the Akaike Information Criterion (AIC) as the best-fit likelihood model in ModelFinder (Kalyaanamoorthy et al. 2017; Zhang et al. 2020). The models employed for the four genes in BI analyses were GTR + F + I + G4 for nrLSU and SYM + I + G4 for *tef1-α*, *rpb1*, and *rpb2*. Subsequently, the sampled trees were summarized after omitting the first 25% of trees as burn-in using the ‘sump’ and ‘sumt’ commands implemented in MrBayes. For BI analyses, the average standard deviation of split frequencies was 0.007272, and all ESS values were > 200 (detailed in Suppl. material 4).

### ﻿Abbreviations

**AIC** the Akaike Information Criterion

**AU test** Approximately Unbiased test

**BI** Bayesian inference

**BP** bootstrap proportion

**BPI** USDA United States National Fungus Collections

**CTAB** hexadecyl trimethyl ammonium bromide, cetyltrimethylammonium bromide

**DNA** deoxyribonucleic acid

**FH** Harvard University, Farlow Herbarium

**ILD** the incongruence length difference

**K** Royal Botanic Gardens,Kew

**KUN-HKAS** The Herbarium of Kunming Institute of Botany, Chinese Academy of Sciences

**ML** Maximum likelihood

**nrLSU** the nuclear ribosomal large subunit

**NY** New York Botanical Garden

**PCR** Polymerase chain reaction

**PH** Academy of Natural Sciences of Drexel University

**PP** posterior probabilities

***rpb1*** largest subunit of RNA polymerase II

***rpb2*** second-largest subunit of RNA polymerase II

***tef1-α*** translation elongation factor 1-α gene

**UPS** Uppsala University, Museum of Evolution

## ﻿Results

### ﻿Phylogenomic analysis

**Figure 1.**
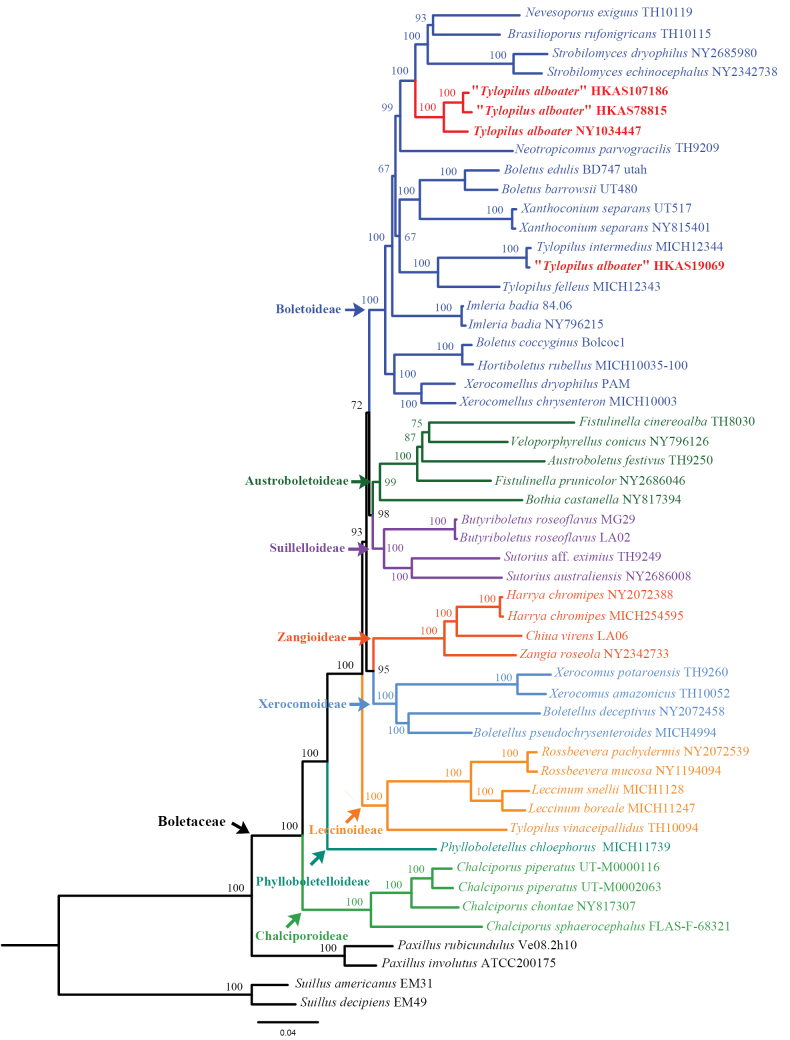
Phylogenomics of the family *Boletaceae*. The tree was inferred from a matrix containing 259 single-copy orthologous genes based on the maximum-likelihood (ML) analysis. The eight subfamilies are highlighted in different colors. Specimens identified as “*Tylopilus alboater*” are in bold and highlighted in red.

Our phylogenomic analysis incorporated 52 high-quality genomes, including 48 representing 42 species from 27 genera across all eight *Boletaceae* subfamilies. Additionally, two *Suillus* of *Suillaceae* and two *Paxillus* of *Paxillaceae* were included as outgroup taxa. All selected genomes met the quality threshold of ≥ 80% BUSCO completeness scores (detailed metrics provided in Suppl. material 1). Using BUSCO assessments, we identified 259 single-copy orthologous genes present in all 52 genomes, which were subsequently selected for phylogenomic analysis. The final alignment comprised 89,095 amino acid sites. Our analysis yielded a robust phylogeny of *Boletaceae* (Fig. 1), unequivocally validating the eight-subfamily classification framework previously proposed by Tremble et al. (2024) and Wu et al. (2023, preprint). However, a minor discrepancy was observed in the phylogenetic placement of *Leccinoideae*, potentially attributable to either the limited number of single-copy orthologous genes analyzed or methodological constraints in phylogenetic reconstruction. Notably, our phylogenomic analysis demonstrated that specimens morphologically identified as *T. alboater* exhibit polyphyly and all clustered within the subfamily *Boletoideae*. A particularly significant finding was the identification of a strongly supported (BP = 100%) monophyletic lineage comprising three specimens: one from New York (NY 1034447) and two from China (HKAS 107186, HKAS 78815). This distinct lineage likely represents a previously unrecognized genus within *Boletoideae*. Additionally, another New York specimen (HKAS 19069) was found to cluster within *Tylopilus*. To comprehensively resolve the phylogenetic relationships of species identified as *T. alboater*, we performed a multi-locus phylogenetic analysis based on four commonly used genes (nrLSU, *tef1-α*, *rpb1*, and *rpb2*), with representatives from all 23 genera of *Boletoideae* to which the *T. alboater* complex belongs, based on our study and that of Tremble et al. (2024).

### ﻿Phylogenetic analysis

**Figure 2.**
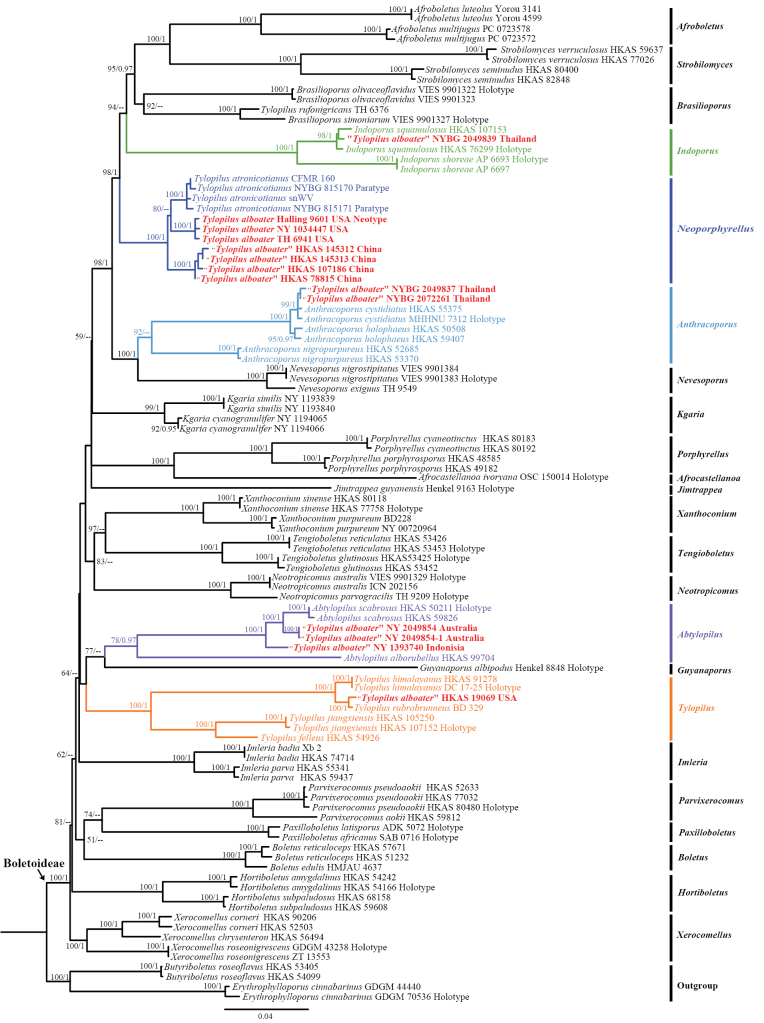
Phylogenetic positions and relationships of species originally identified as “*T. alboater*.” Samples from Asia, North America, and Australia were inferred from a multi-locus dataset (nrLSU + *rpb1* + *rpb2* + *tef1-α*) using the maximum-likelihood (ML) and Bayesian inference (BI) methods (only the ML tree is shown). Bootstrap frequencies (BP > 50%) and posterior probabilities (PP > 0.95) are shown above supported branches. Specimens identified as *T. alboater* are highlighted in red and belong to five different colored generic lineages. Vouchers and localities are provided after the species names.

The combined dataset (nrLSU + *tef1-α* + *rpb1* + *rpb2*), including 100 sequences representing all 23 genera of *Boletoideae* and two genera of *Suillelloideae*, consisted of 3,056 nucleotides (including gaps) with the following gene lengths: nrLSU, 902 bp; *tef1-α*, 640 bp; *rpb1*, 806 bp; and *rpb2*, 708 bp (Suppl. material 5). The alignment was submitted to TreeBASE (S30298). In our multi-locus phylogenetic analyses, both ML and BI approaches produced highly congruent tree topologies, with minimal discrepancies observed in branch support values; thus, only the ML tree was selected for display (Fig. 2). Our multi-locus phylogenetic analyses revealed topological discrepancies compared to both the phylogenomic results of Tremble et al. (2024) and our current study. Specifically, the terminal genus-level branches received statistical support, whereas several backbone branches lacked sufficient support. This is potentially attributable to the limited number of genes analyzed.

Thirteen samples were studied, including two (NY 815170, NY 815171) of *T. atronicotianus*, both from North America, and eleven labeled as *T. alboater* from Australia, China, Indonesia, Thailand, and the USA. Sequences of these samples represent eight species and are nested within five generic lineages of the subfamily *Boletoideae*, including one new genus, *Neoporphyrellus*, with one new species (*N. sinoalboater* from China) and two new combinations (*N. alboater* and *N. atronicotianus*, both from the USA). The other five species are nested in four known genera, including two new species of *Abtylopilus* (*Ab. australiensis* from Australia and *Ab. indonesiensis* from Indonesia) and three known species belonging to *Anthracoporus* (*An. cystidiatus* from Thailand), *Indoporus* (*I. squamulosus* from Thailand), and *Tylopilus* (*T. rubrobrunneus* from the USA), respectively.

### ﻿Morphologic observations

Seven samples represent three species, including one new species (*N. sinoalboater*) and two new combinations (*N. alboater* and *N. atronicotianus*) of the newly proposed genus *Neoporphyrellus*. The new species *N. sinoalboater*, with three samples (KUN-HKAS 107186, KUN-HKAS 145312, KUN-HKAS 145313) from Yunnan Province, southwest China, and another sample (KUN-HKAS 78815) from Hubei Province, central China, is characterized by its gray to brownish gray pileus and stipe staining reddish brown at first and then becoming blackish when bruised; white to cream hymenophore usually staining reddish and then blackish when bruised; white to pallid context changing to reddish or brownish red initially and then slowly becoming blackish when injured; smooth basidiospores; and trichoderm pileipellis. For the new combination *N. alboater*, representing our inferred concept of *T. alboater* sensu stricto, one sample (NY 1193926) was analyzed from among the 21 gathered by R.E.H. (second author) over 35 years (1984–2019) on the 100-ha property of the New York Botanical Garden, northeastern USA. That site is approximately 150 km east of the type locality of *T. alboater*. *Neoporphyrellus alboater* is characterized by its black to dark gray to dark brown pileus, which is areolate with age; white context slowly turning light pinkish orange or red to pinkish gray, then black when injured; finely subpruinose stipe that is white above and black below at first and eventually black overall; white to pinkish vinaceous hymenophore becoming pinkish orange to reddish brown then black when bruised; smooth basidiospores; and trichoderm pileipellis. For the other combination, *N. atronicotianus*, it is characterized by its light brown to olive-brown pileus; whitish context staining pink to pinkish red, then becoming black when injured; whitish hymenophore staining red at first and then blackish when injured; smooth basidiospores; and trichodermium pileipellis.

The remaining six samples represent five species belonging to *Abtylopilus*, *Anthracoporus*, *Indoporus*, and *Tylopilus*. Two samples from Indonesia (NY 1393740) and Australia (NY 2049854) represent two new species of *Abtylopilus* proposed as *Ab. australiensis* and *Ab. indonesiensis*, respectively. *Abtylopilus australiensis* is characterized by the dark brown pileus, the cream to reddish hymenophore becoming orange red with age, the reddish brown to blackish stipe that is cream to yellowish and then orange red to brownish red when mature at the apex, and the densely minute-pruinose stipe surface. *Abtylopilus indonesiensis* is characterized by the chocolate brown to black pileus becoming darker when bruised, the pinkish hymenophore staining orange red initially then blackish when bruised, and the deep purple to black stipe that is white to pinkish orange at the apex. Three samples from Thailand represent *I. squamulosus* (NY 2049839) and *An. cystidiatus* (NY 2049837 and NY 2072261), respectively. *Indoporus squamulosus*, originally described by Li and Yang (2021) from China, is characterized by the black to blackish or gray pileus, the extended pileal margin, the white to grayish white or gray hymenophore, the initially red and then black discoloration when injured, the palisadoderm pileipellis, and the smooth basidiospores. *Anthracoporus cystidiatus*, also originally described by Li and Yang (2021) from China, is characterized by the grayish red to brownish red or ruby red pileus, the black to grayish pink hymenophore, the white to pallid context, the fine hymenophoral pores, the initially red and then black discoloration when injured, the epithelial pileipellis composed of 8–21 μm wide inflated concatenated cells, and the smooth basidiospores. One sample (KUN-HKAS 19069) from the USA represents *T. rubrobrunneus*, originally described by Mazzer and Smith (1967) from the USA, and is characterized by the vinaceous brown to chocolate pileus, a whitish hymenophore becoming vinaceous with age, white context staining olivaceous when injured, a trichoderm pileipellis composed of 4–6 μm wide loosely interwoven hyphae, and smooth basidiospores. The new taxa delimited in this study are illustrated and documented herein.

### ﻿Taxonomy

#### ﻿ Neoporphyrellus

Taxon classificationAnimaliaBoletalesBoletaceae

Yan C. Li, J. Li, Halling, Osmundson & Zhu L. Yang gen. nov.

F889B215-69B9-53FE-A8B9-F7D1E99AD40B

858938

##### Diagnosis.

This genus differs from other genera of *Boletaceae* in its dark colored basidioma, white to cream and thin hymenophore at first, then pinkish to light gray, white mycelium on the base of stipe, trichoderm pileipellis, smooth basidiospores, and initially reddish then blackish discoloration in the context when injured.

##### Etymology.

*Neoporphyrellus* (Latin) reflecting the new genus shares the similar colors of the basidiomata and spore prints with the genus *Porphyrellus*.

##### Typus generis.

*Neoporphyrellus alboater* (Schwein.) Yan C. Li, J. Li, Halling, Osmundson & Zhu L. Yang [Basionym: *Boletus alboater* Schwein.].

##### Description.

Basidiomata small to large-sized. Pileus subhemispherical, broadly convex to applanate; surface dry, smooth or with a velvet-like texture, cracked with age, gray to brownish gray, or light brown to olive-brown, or yellowish gray to orangish brown. Context whitish, staining pink to pinkish red or reddish or brownish red at first and then becoming blackish when injured. Hymenophore slightly depressed around apex of stipe; surface initially whitish to cream then yellowish or grayish when mature, changing to reddish at first and then becoming blackish when injured; pores roundish or angular to irregular, changing to reddish or reddish brown at first and then becoming blackish when injured. Stipe clavate to subcylindrical, flexuous, solid, grayish to blackish brown, or yellowish brown, brownish black to black, dark in color downwards, sometimes slightly reticulate near apex; context white to cream, changing to brownish red quickly, then slowly becoming blackish when injured. Taste and odor mild. Basidiospores smooth, subfusiform. Cheilo- and pleurocystidia fusiform or subfusiform. Pileipellis trichodermium composed of somewhat vertically arranged or interwoven thin-walled hyphae. Clamp connections absent in all tissues.

##### Note.

The genus *Neoporphyrellus* is characterized by the gray to purplish gray pileus, finely velvety pileal surface, initially white to cream and thin hymenophore becoming pinkish vinaceous, white context changing reddish initially and then blackish when injured, and trichoderm pileipellis. Some recently published new genera, viz. *Abtylopilus*, *Anthracoporus*, *Brasilioporus*, *Indoporus*, *Kgaria*, and *Nevesoporus*, were proposed based on species that were thought to belong to, or be closely related to *Tylopilus* and have a context changing reddish initially and then blackish when injured, using both morphological and molecular methods (Parihar et al. 2018; Li and Yang 2021; Magnago et al. 2022; Halling et al. 2023). However, *Abtylopilus* is characterized by its nearly glabrous pileus, white to cream hymenophore, fine hymenophoral pores (0.3–1 mm wide), and palisadoderm pileipellis composed of 4–11 μm wide, vertically arranged hyphae (Li and Yang 2021). *Anthracoporus* is characterized by the tomentose or rugose pileus, black to grayish black hymenophore when young and then becoming grayish pink when mature, fine hymenophoral pores (0.3–2 mm wide), and trichoderm pileipellis composed of 4–9.5 μm hyphae, or an epithelial pileipellis composed of 10–21 μm wide inflated concatenated cells (Li and Yang 2021). *Brasilioporus* is characterized by the matted fibrillose to squamulose pileus, the off-white hymenophore and context when young and then pinkish with age, fine hymenophoral pores (1–1.5 mm wide), and a trichoderm pileipellis with terminal cells 8–12 μm wide (Magnago et al. 2022). *Indoporus* is characterized by its distinct extended pileal margin, gray or grayish white to grayish pink hymenophore, large hymenophoral pores (up to 4 mm wide), and trichoderm to palisadoderm pileipellis composed of 6–16 μm wide, vertically arranged hyphae (Parihar et al. 2018; Li and Yang 2021). *Kgaria* is characterized by its irregularly bumpy to roughened pileus, sometimes scaly-areolate with age, white context changing from blue to red and then nearly black, white then mineral green to dull yellow to olive-brown hymenophore changing from red to blue and then black when bruised, and trichoderm pileipellis and stipitipellis with iconic cyanogranular encrusting pigment (Halling et al. 2023). *Nevesoporus* is characterized by the initially velvety and then subareolate pileus, the relatively large hymenophoral pores (1–2 mm wide), the off-white hymenophore when young and then becoming pinkish when mature, unchanging or blackish when bruised, and the trichoderm pileipellis consisting of vertically arranged catenulate hyphae (Magnago et al. 2022).

*Porphyrellus* shares similarly colored spore prints and basidiomata with *Neoporphyrellus*, but it can be distinguished from *Neoporphyrellus* by its white to pallid context without discoloration or becoming asymmetrically blue or at first reddish and then bluish when injured, and its palisadoderm or epithelial pileipellis (Gilbert 1931; Wolfe 1979; Li and Yang 2021). Currently, three species of *Neoporphyrellus* are revealed including one new species and two new combinations. These three species are documented and illustrated below:

#### ﻿ Neoporphyrellus alboater

Taxon classificationAnimaliaBoletalesBoletaceae

(Schwein.) Yan C. Li, J. Li, Halling, Osmundson & Zhu L. Yang comb. nov.

1424CB60-A5FE-5B71-A469-BBE3FE91903C

858940

Figs 3, 8a, b

Suillus alboater (Schwein.) Kuntze, Revis. gen. pl. (Leipzig) 3(3): 535. 1898.
Tylopilus alboater (Schwein.) Murrill, Mycologia 1(1): 16. 1909.
Porphyrellus alboater (Schwein.) E.-J. Gilbert, Les Bolets: 99. 1931.

##### Basionym.

*Boletus alboater* Schwein., Schr. naturf. Ges. Leipzig 1: 95. 1822.

##### Description.

Basidiomata medium to large sized. Pileus 4.5–13.5 cm broad, convex to plano-convex to plane, dry, subvelutinous to matte, black to dark gray to dark brown, becoming gray, becoming finely areolate with age, with even and entire margin; context white, up to 3 cm thick, slowly light pinkish orange or red to pinkish gray, then black when injured, with mild odor and taste. Hymenophore adnate to adnexed; surface white at first becoming pinkish vinaceous, staining pinkish orange to reddish brown then black when injured; tubes up to 10 mm long. Stipe 5–9 × 1–3 cm, equal to subclavate, dry, finely subpruinose, sometimes obscurely ridged below, white above and black below at first, eventually black overall, white to pale grayish at base; context white above, gray to black below, becoming black with age. Spore print pinkish vinaceous. Taste and odor mild (Coker and Beers 1943; Singer 1947; Snell and Dick 1970; Smith and Thiers 1971; Roody 2003; Phillips 2005; Moore et al. 2007; Bessette et al. 2024; our observation).

Basidia 20–31 × 9.5–12 μm, clavate, thin-walled, 4-spored, hyaline to yellowish in KOH. Basidiospores [60/2/1] (8) 8.5–10.5 (11) × 4–5 μm, [Q = (1.78) 1.89–2.63 (2.75), Q_m_ = 2.18 ± 0.17], subfusiform in profile view with slight suprahilar depression, elongated to fusiform in ventral view, smooth, slightly thick-walled (up to 0.5 μm), hyaline to brownish in KOH, brown to yellowish brown in Melzer’s reagent. Hymenophoral trama boletoid; hyphae cylindrical, hyaline to yellowish in KOH, yellowish to yellow in Melzer’s reagent. Cheilocystidia 24–46 × 8–18 μm, fusiform or subfusiform, thin-walled, yellowish brown to brownish in KOH, yellow-brown to brown in Melzer’s reagent; surface without encrustations. Pleurocystidia 39–56 × 13–17 μm, morphologically similar to cheilocystidia. Pileipellis a trichoderm, composed of 4–8.5 μm wide filamentous interwoven hyphae, yellowish brown to brown in KOH, and brown to dark brown in Melzer’s reagent; terminal cells 22–58 × 4–6.5 μm, clavate to subcylindrical or fusiform, thin-walled. Pileal trama composed of thin-walled hyphae; hyphae 4.5–9 μm wide, hyaline to yellowish in KOH, brownish to yellowish brown in Melzer’s reagent. Clamp connections absent in all tissues.

##### Habitat and distribution.

Solitary, scattered to gregarious under trees of the genera *Quercus*, *Fagus*, *Betula*, *Carya*; currently known from eastern United States, New York to northern Florida, west to Missouri.

##### Specimen examined.

USA • New York, Bronx Co., Bronx, New York Botanical Garden, River Way & Snuff Mill Rd., ca 20 m, 40.8586°N, 73.8776°W, 13 August 1984, Halling 3794 (NY45157), 19 August 1984, Halling 3803 (NY45194), 5 September 1984, R. E. Halling 3819 (NY45190), 24 July 1989, Halling 6277 (NY45187); Azalea Way, 40.8624°N, 73.8781°W, 10 September 1984, Halling 3841 (NY45189), 7 July 1989, Halling 6246 (NY45197), 9 July 1992, Halling 6866 (NY45196), 1 August 1992, Halling 6883 (NY45182), 40.8624°N, 73.8781°W, 7 July 1985, Halling 4431 (NY45199), 40.8619°N, 73.8777°W, 50 m, 5 August 1985, Halling 4493 (NY45198); near Twin Ponds, 40.8665°N, 73.8754°W, 30 July 1988, Halling 5964 (NY45200), 4 August 1988, Halling 5971 (NY45195), 12 June 1991, Halling 6542 (NY45186), 27 August 1991, Halling 6633 (NY45191); east end of eastern Twin Pond, 40.8671°N, 73.8754°W, 21 August 1985, Halling 4549 (NY14583, NY14584), 8 September 2008, Halling 9013 (NY1034447), 23 August 2011, Halling 9601 (NY1193926 and KUN-HKAS147016); 40.8667°N, 73.8753°W, 35–36 m, 14 July 2016, Halling 10083 (NY02685939), 9 August 2017, Halling 10154 (NY02861405), 3 August 2018, Halling 10176 (NY02072595), 13 September 2019, Halling 10186 (NY02072672).

##### Typification of the name *Boletus alboater*.

*Neoporphyrellus alboater* was originally described as *Boletus alboater* from Bethlehem, Pennsylvania, USA, by Schweinitz (1822). Since then, typification has not been verified, even though #864 was the number applied to a specimen by Schweinitz. We have tried to locate the original material in PH for lectotypification but without success. Also, we have checked K, FH, BPI, and UPS but failed to find that specimen. In PH, there is another specimen (PH00078766) that was collected by Persoon and verified by Schweinitz as *Boletus alboater*. Persoon clearly did not collect in the USA, so it is highly unlikely that the identification is correct. Also, based on current knowledge, *B. alboater* has not been documented in Europe. According to information listed on MycoPortal.org, another specimen in PH (00078756) listed as *Boletus* sp., determined by Schweinitz, with an “ID Remarks” as “*Boletus alb.*” The collector is listed as unknown, there are no locality data, and a verbatim date of “1805-01-02” is cited without a known collector number. Thus, due to a lack of valid original material, we have proposed here a neotype in NY with an isoneotype in KUN. As we noted above, the NYBG site is only ± 150 km east of the type locality cited later by Schweinitz (1832).

##### Type.

**Figure 3.**
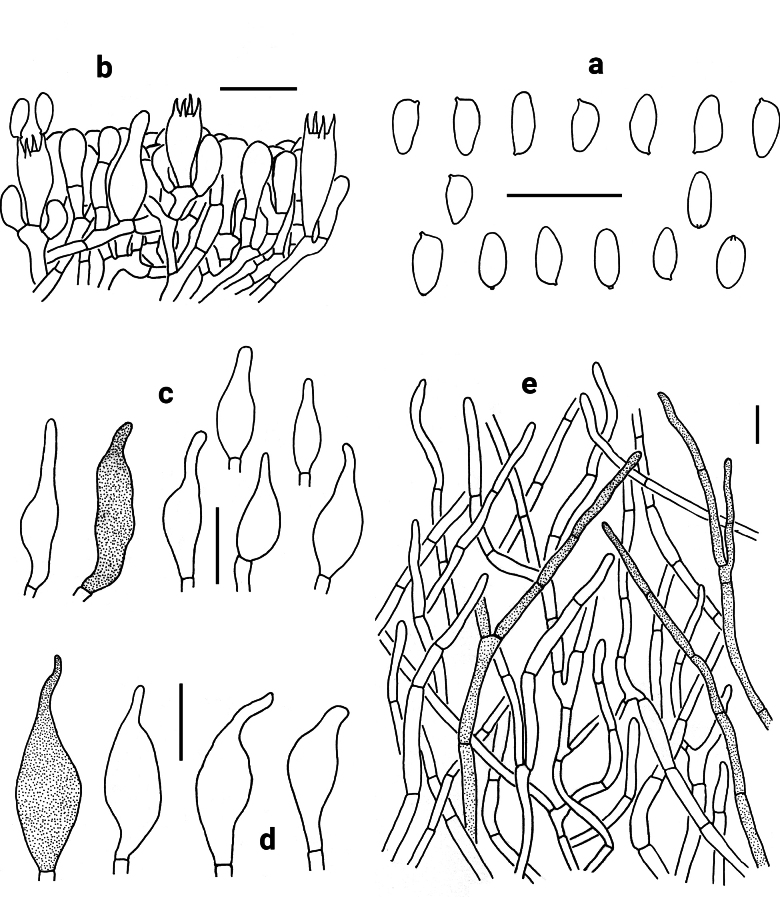
Microscopic features of *Neoporphyrellus alboater* (NY 1193926). **a** Basidiospores **b** Basidia, basidioles and pleurocystidium **c** Cheilocystidia **d** Pleurocystidia **e** Pileipellis. Scale bars: 20 μm.

**(Neotype designated here**, Figs 3, 7a, b, Mycobank No.: MBT 10026353): USA • New York, Bronx Co., Bronx, New York Botanical Garden, east end of eastern Twin Pond, 23 August 2011, Halling 9601 (Neotype: NY1193926, GenBank Acc. Nos. OP771514 for nrLSU, OP765296 for *rpb1*, OP762644 for *rpb2*, OP750052 for *tef1-α*; Isoneotype: KUN-HKAS147016).

##### Notes.

*Neoporphyrellus alboater* is characterized by its black to dark gray to dark brown pileus, which is areolate with age; white context slowly turning light pinkish orange or red to pinkish gray, then black when injured; finely subpruinose stipe, which is white above and black below at first and eventually black overall; white to pinkish vinaceous hymenophore becoming pinkish orange to reddish brown, then black when bruised; smooth basidiospores; and trichoderm pileipellis (Coker and Beers 1943; Singer 1947; Snell and Dick 1970; Smith and Thiers 1971; Roody 2003; Phillips 2005; Moore et al. 2007; Bessette et al. 2024). This species shares basidiospore size and the same discoloration reaction when injured with *An. cystidiatus* (Li and Yang 2021). However, *An. cystidiatus* differs from *N. alboater* in its grayish red to brownish red or ruby red pileus, which is slightly darker in the center, and its epithelial pileipellis composed of 8–21 μm wide inflated concatenated cells. The white hymenophore when young and dull pink when mature and the sometimes slightly reticulated stipe apex of *N. alboater* are similar to those of *N. atronicotianus*. Moreover, both of these species are phylogenetically related. However, *N. atronicotianus* has a finely tomentose pileus and stipe, a bright brown hymenophore, a reddish-brown spore print, relatively large basidia (31–46 × 7.5–9.5 μm), and narrow pleurocystidia and cheilocystidia (9–12 μm and 6–9 μm wide, respectively).

#### ﻿ Neoporphyrellus atronicotianus

Taxon classificationAnimaliaBoletalesBoletaceae

(Both) Yan C. Li, J. Li, Halling, Osmundson & Zhu L. Yang comb. nov.

2E167ECE-A304-539D-910A-11D86D2C5CD9

858941

Figs 4, 8c, d

##### Basionym.

*Tylopilus atronicotianus* Both, Bull. Buffalo Soc. Nat. Sci. 36: 216. 1998

##### Description.

Basidiomata medium to large sized. Pileus 7.5–20 cm in diameter, hemispherical to broadly convex or flattened; surface dry, smooth, light brown to olive-brown, becoming darker in color when matured; context whitish, staining pink to pinkish red at first, and then becoming black when injured. Hymenophore surface initially whitish, staining red at first and then blackish when injured; pores angular, up to 1.5 mm wide; tubes up to 8 mm long, bright brown, staining reddish at first and then black when injured. Stipe 6–12 × 1.5–4.5 cm, clavate to subcylindrical, solid, grayish to dark brown, almost black at the base, surface finely tomentose, sometimes finely reticulate near apex; context white, pink to pinkish-red at first, and then becoming black when injured. Spore print reddish-brown (Both 1998; Bessette et al. 2000, 2024; and our observation).

Basidia 31–46 × 7.5–9.5 μm, clavate, thin-walled, 4-spored, hyaline to yellowish in KOH. Basidiospores [40/2/2] 7.5–10.5 × 3.5–5 μm [Q = 1.78–2.38, Q_m_ = 2.14 ± 0.09], subfusiform in profile view with slight suprahilar depression, elongated to fusiform in ventral view, smooth, slightly thick-walled (up to 0.5 μm), hyaline to brownish in KOH, brown to yellowish brown in Melzer’s reagent. Hymenophoral trama boletoid; hyphae cylindrical, hyaline to yellowish in KOH, yellowish to yellow in Melzer’s reagent. Cheilocystidia 32–47 × 6–9 μm, fusiform or subfusiform, thin-walled, yellowish brown to brownish in KOH, yellow-brown to brown in Melzer’s reagent; surface without encrustations. Pleurocystidia morphologically similar to cheilocystidia but much bigger, 38–76 × 9–12 μm. Pileipellis a trichoderm, composed of 3.5–7 μm wide filamentous interwoven hyphae, yellowish brown to brownish in KOH, and brown to yellow-brown in Melzer’s reagent; terminal cells 11.5–76 × 3.5–5.5 μm, clavate to subcylindrical, thin-walled. Pileal trama composed of thin-walled hyphae; hyphae 4.5–9 μm wide, hyaline to yellowish in KOH, yellow to yellowish brown in Melzer’s reagent. Clamp connections absent in all tissues.

**Figure 4.**
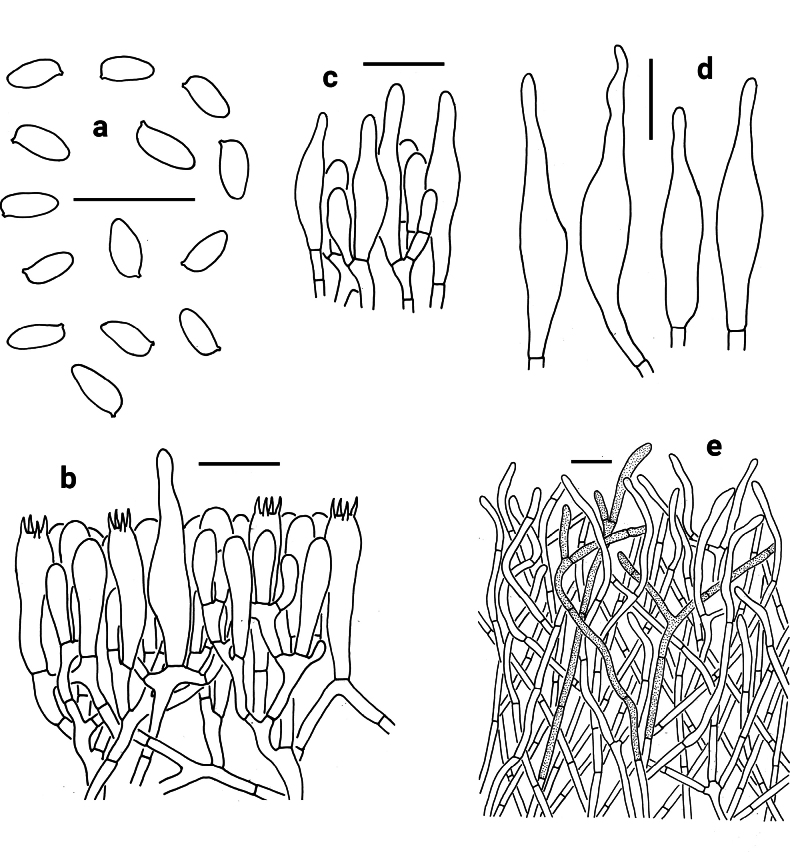
Microscopic features of *Neoporphyrellus atronicotianus* (NY 00815170, paratype). **a** Basidiospores **b** Basidia, basidioles and pleurocystidium **c** Cheilocystidia **d** Pleurocystidia **e** Pileipellis. Scale bars: 20 μm.

##### Habitat and distribution.

Solitary on the ground of deciduous forest dominated by red oak (*Quercus rubra*), beech (*Fagus* sp.), and hemlock (*Tsuga* sp.); currently known in the United States from New York to West Virginia.

##### Specimen examined.

USA • New York, Erie Co., North Collins, Town Park, alt. ca 1319 m, 42.5953°N, 78.9411°W, August 1983, E.E. Both 2480 (NY 815170, paratype); New York, Erie Co., North Collins, Ukrainian Camp, alt. ca 1319 m, 42.5953°N, 78.9411°W, 18 September 1981, B. Both 2358 (NY 815171, paratype).

##### Notes.

*Neoporphyrellus atronicotianus* was originally described from New York and is currently known from the eastern USA (Both 1998; Bessette et al. 2000, 2024). It is characterized by its light brown to olive-brown pileus, whitish context staining pink to pinkish-red and then becoming black when injured, whitish hymenophore staining red at first and then blackish when injured, smooth basidiospores, and trichodermium pileipellis (Both 1998; Bessette et al. 2000, 2024; and our observation). This species is morphologically similar to *T. alpinus* Yan C. Li & Zhu L. Yang, as both of them have an olive-brown pileus, whitish context, whitish hymenophore, and reticulum at the upper part of the stipe. However, *T. alpinus* differs from *N. atronicotianus* in its context staining pale red to grayish red but without any black or blackish tinges when injured, hymenophore staining a brownish red to grayish red or orange-brown tinge when bruised, and relatively long basidiospores (13–14.5 μm) (Li and Yang 2021). *Neoporphyrellus atronicotianus* is phylogenetically closely related to *N. alboater* and *N. sinoalboater*. However, *N. alboater* has a black to dark gray to dark brown pileus, which is areolate with age, a finely subpruinose stipe, a white to pinkish vinaceous hymenophore, and relatively broad cheilocystidia measuring 24–46 × 8–18 μm and pleurocystidia measuring 39–56 × 13–17 μm. While *N. sinoalboater* differs from *N. atronicotianus* in its gray to brownish gray pileus and relatively small pleurocystidia (34–55 × 8–14 μm).

#### ﻿ Neoporphyrellus sinoalboater

Taxon classificationAnimaliaBoletalesBoletaceae

Yan C. Li, J. Li, Halling, Osmundson & Zhu L. Yang sp. nov.

9EA478B7-D0C7-5CEE-B442-3504C12994F8

858942

Figs 5, 8e, f

##### Etymology.

*sino* (Latin) = China, reflecting that the basidiomata were collected from China + *alboater* for the similarity of the basidiomata to *T. alboater*.

##### Type.

CHINA • Yunnan Province, Lijiang City, Liming Town, Laojunshan, alt. ca 2516 m, 26.8470°N, 99.8502°E, 31 August 2024, Y.C. Li 6989 (KUN-HKAS 145312).

##### Diagnosis.

*Neoporphyrellus sinoalboater* differs from other species of *Neoporphyrellus* in its gray to brownish gray pileus, whitish to pallid context, whitish to cream and then dirty white or grayish hymenophore, grayish to blackish brown stipe, a trichoderm pileipellis composed of 3.5–7 μm wide filamentous interwoven hyphae.

##### Description.

Basidiomata medium to large sized. Pileus 3–4.5 cm in diameter, subhemispherical to applanate; surface dry, smooth, gray (5B1) to brownish gray (5B2), staining reddish brown when bruised; margin slightly extended; context whitish (1A2) to pallid (1A3), changing to reddish (8B4) or brownish red (8C4) quickly, and then slowly becoming blackish (19F5) when injured. Hymenophore adnate to slightly decurrent, or sometimes slightly depressed around apex of stipe when mature; surface initially whitish (1A2) to cream (1A3) or dirty white to grayish (4B1); pores angular to roundish, up to 1 mm wide, tubes up to 6 mm long, concolorous or a little paler than hymenophoral surface, changing to reddish brown (8C3) at first and then becoming blackish when injured. Stipe 5–8.3 × 1–4 cm, clavate to subcylindrical, flexuous, solid, grayish (5C3) to blackish brown (5E2), dark in color downwards, staining reddish brown at first and then blackish when bruising; context white (1A1) to cream (1A2), changing to brownish red (8C4) quickly, then slowly becoming blackish (19F5) when injured; basal mycelium white (1A1). Taste and odor mild.

**Figure 5.**
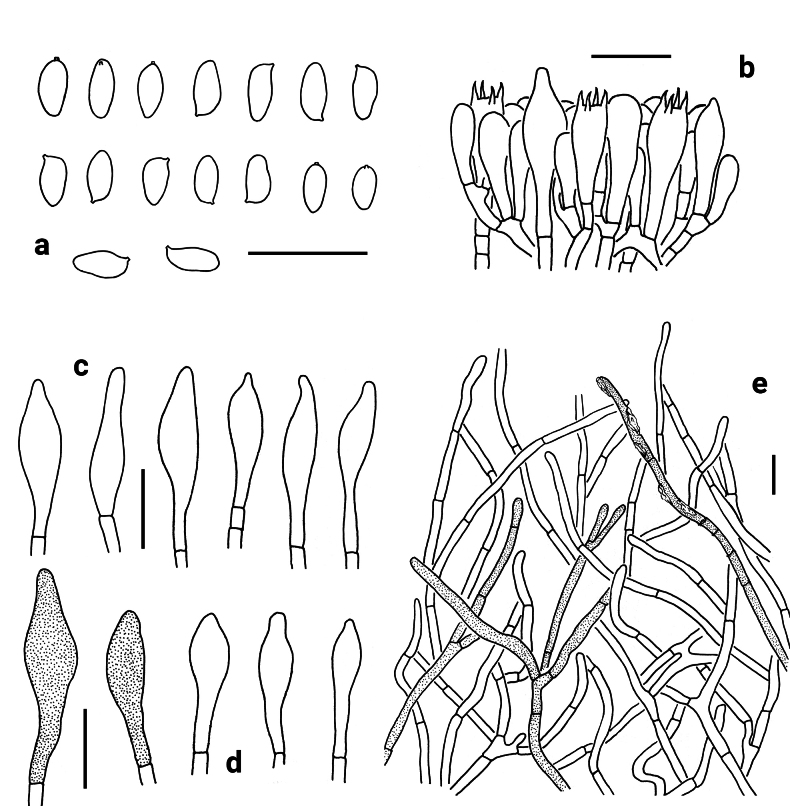
Microscopic features of *Neoporphyrellus sinoalboater* (KUN-HKAS107186). **a** Basidiospores **b** Basidia, basidioles and pleurocystidia **c** Pleurocystidia **d** Cheilocystidia; **e** Pileipellis. Scale bars: 20 μm.

Basidia 25–34 × 9–10 μm, clavate, thin-walled, 4-spored, hyaline or yellowish brown in KOH. Basidiospores [80/4/4] (7) 7.5–9.5 (10) × (3.5) 4–4.5 (5) μm, [Q = (1.67) 1.75–2.25 (2.38), Q_m_ = 1.99 ± 0.13], subfusiform in profile view with slight suprahilar depression, elongated to fusiform in ventral view, smooth, slightly thick-walled (up to 0.5 μm), yellowish or brownish in KOH, brown to yellowish brown in Melzer’s reagent. Hymenophoral trama boletoid; hyphae cylindrical, 4–10 μm wide, hyaline to yellowish in KOH, yellow to yellowish brown in Melzer’s reagent. Cheilocystidia 34–55 × 8–14 μm, fusiform or subfusiform, thin-walled, hyaline to brownish in KOH, yellowish brown to brownish in Melzer’s reagent; surface without encrustations. Pleurocystidia morphologically similar to cheilocystidia. Pileipellis a trichoderm, composed of 3.5–7 μm wide filamentous interwoven hyphae, yellowish brown to brownish in KOH and brown to dark brown in Melzer’s reagent; terminal cells 15–88 × 3.5–7 μm, clavate to subcylindrical or fusiform, thin-walled. Pileal trama composed of thin-walled hyphae, 3.5–6 μm wide, hyaline or yellowish in KOH, brownish in Melzer’s reagent. Clamp connections absent in all tissues.

##### Habitat and distribution.

Solitary on the ground under *Quercus semicarpifolia*; currently known from central and southwestern China.

##### Additional specimens examined.

CHINA • Hubei Province, Shennongjia, Muyu Town, alt. ca 1800 m, 31.4689°N, 110.3663°E, 16 July 2012, Q. Zhao1556 (KUN-HKAS 78815); Yunnan Province, Dali, Xiangyun Town, Dasongping Village, alt. ca 2040 m, 25.6653°N, 100.6955°E, 10 July 2009, N.K. Zeng 297 (KUN-HKAS 107186); Lijiang City, Liming Town, Laojunshan, alt. ca 2516 m, 26.8470°N, 99.8502°E, 31 August 2024, Y.C. Li 7010 (KUN-HKAS 145313).

##### Note.

*Neoporphyrellus sinoalboater* is characterized by its gray to brownish gray pileus and stipe staining reddish brown at first and then becoming blackish when bruised; white to cream hymenophore usually staining reddish and then blackish when bruised; white to pallid context changing to reddish or brownish red initially and then slowly becoming blackish when injured; smooth basidiospores; and trichoderm pileipellis. All these features are very similar to those of *Abtylopilus scabrosus* Yan C. Li & Zhu L. Yang and *Abtylopilus alborubellus* Yan C. Li & Zhu L. Yang. However, the latter two species have a glabrous pileus, a white to cream or grayish and then grayish pink hymenophore, relatively long basidiospores (up to 11 μm), and a palisadoderm pileipellis composed of broad (up to 9 μm) vertically arranged hyphae (Li and Yang 2021). *Neoporphyrellus alboater* and *N. atronicotianus* share the same discoloration as *N. sinoalboater* when injured, but *N. alboater* has a black to dark gray to dark brown pileus becoming gray with age; a dull pinkish or flesh-colored hymenophore; fine hymenophoral pores (up to 0.5 mm wide); relatively large basidiospores measuring 8–11 × 4–5 μm; and wide hymenial cystidia measuring 39–56 × 13–17 μm (Singer 1947). *Neoporphyrellus atronicotianus* has a light brown to olive-brown pileus, a grayish to dark brown stipe with the base almost black, relatively long tubes (up to 22 mm), and relatively large basidia measuring 31–46 × 7.5–9.5 μm (Singer 1947; Bessette and Bessette 2000; Bessette and Bessette 2001; Roody 2003).

#### ﻿ Abtylopilus

Taxon classificationAnimaliaBoletalesBoletaceae

Yan C. Li & Zhu L. Yang, The Boletes of China: Tylopilus s. l. (Singapore): 39 (2021)

741186B0-DC1C-50D6-B0F2-68A14405DCB7

##### Notes.

*Abtylopilus* was proposed by Li and Yang (2021) as a new genus based on morphological and multi-locus phylogenetic studies. This genus is characterized by its nearly glabrous pileus, white to cream hymenophore, fine hymenophoral pores (0.3–1 mm wide), initially red and then black discoloration when injured, and palisadoderm pileipellis. Two species were described from China, viz., *Ab. alborubellus* and *Ab. scabrosus*. Here two additional new species from Indonesia and Australia are described and documented in detail.

#### ﻿ Abtylopilus australiensis

Taxon classificationAnimaliaBoletalesBoletaceae

Yan C. Li, J. Li, Halling, Osmundson & Zhu L. Yang sp. nov.

F0E93B91-F103-5BBE-94C8-675708BAD025

859185

Figs 6, 8g

##### Etymology.

*australiensis* referring to the species being found in Australia.

##### Type.

AUSTRALIA • Queensland, Tablelands, Mareeba, Barron Gorge National Park, Wright’s Lookout, 18.8401°S, 145.6427°W, ca 367 m, 4 Feb 2006, T.W. Osmundson 1080 (Holotype: BRI AQ0796293, Isotype: NY 2049854).

##### Diagnosis.

*Abtylopilus australiensis* differs from other species of *Abtylopilus* in its reddish-brown pileus, reddish to orange-red hymenophore staining dark red initially then blackish when bruised, orange-red, brownish red to blackish stipe, and a trichoderm pileipellis composed of 3–4 μm wide vertically arranged to slightly interwoven hyphae.

##### Description.

Basidiomata medium to large sized. Pileus 3.4–5.6 cm in diameter, convex to plane; surface subtomentose, dry, reddish brown(7E7-7F7); context whitish, staining dark red at first, and then becoming black when injured. Hymenophore depressed around apex of stipe; surface cream (4A3-4A4) to reddish (6A3-6A4), becoming orange-red (7A6-7A7) in age, staining dark red initially then blackish when bruised; pores nearly round, 0.3–1 mm wide, pale cream(4A2), becoming pale pinkish brown (7D5) to nearly black(4F6) in age; tubes up to 4 mm long, concolorous with hymenophoral surface. Stipe 4–5.9 × 0.7–1.1 cm, equal, solid, cream (4A2) to yellowish (4A4) when young, orange-red (7A6) to brownish red (7D8) at apex and reddish brown (6D8-6E8) to blackish (4F6) downward when mature; surface densely covered with minute-pruinose squamules; context whitish, staining dark red at first, and then becoming black when injured.

**Figure 6.**
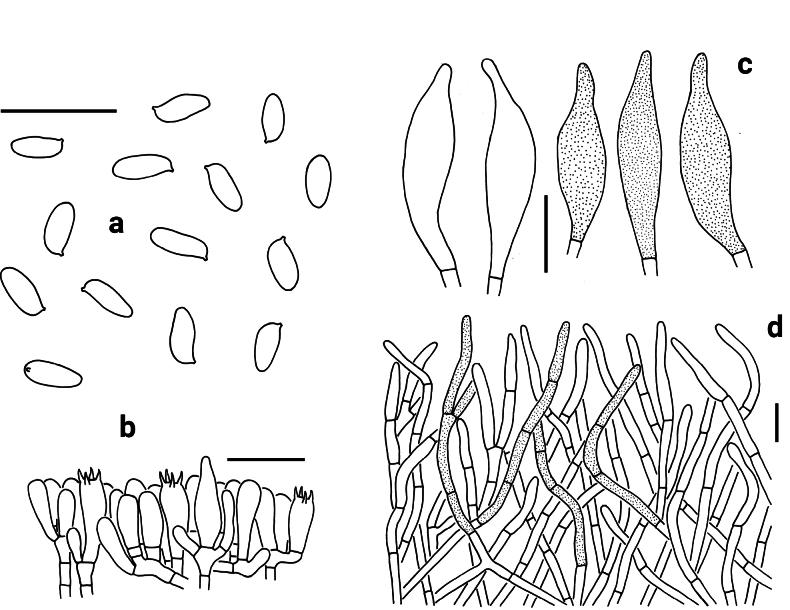
Microscopic features of *Abtylopilus australiensis* (NY 2049854, type). **a** Basidiospores **b** Basidia and pleurocystidium **c** Cheilo- and pleurocystidia **d** Pileipellis. Scale bars: 20 μm.

Basidia 25–30 × 8–12 μm, clavate to narrowly clavate, thin-walled, 4-spored, hyaline to yellowish in KOH. Basidiospores [60/2/1] (8.0) 8.5–10.5 (11) × (3.0) 3.5–4.5 (5) μm [Q = (1.78) 2.11–2.86 (3.17), Q_m_ = 2.47 ± 0.23], subcylindrical or subfusiform and inequilateral in profile view with slight suprahilar depression, oblong to fusiform in ventral view, smooth, yellowish to brownish in KOH, yellow to yellow-brown in Melzer’s reagent. Hymenophoral trama boletoid; hyphae cylindrical, hyaline to yellowish in KOH, yellowish to yellow in Melzer’s reagent. Cheilocystidia 45–56 × 12–13 μm, broadly subfusiform to fusoid-ventricose, thin-walled, yellowish to brownish in KOH, yellow to yellow-brown in Melzer’s reagent; surface without encrustations. Pleurocystidia morphologically similar to cheilocystidia. Pileipellis a trichoderm, composed of 3–4 μm wide vertically arranged to slightly interwoven hyphae, yellowish brown to brown in KOH and yellow-brown to dark brown in Melzer’s reagent; terminal cells 17–27 × 2–3 μm, subfusiform to cystidioid. Pileal trama composed of thin-walled hyphae; hyphae 2.5–4 μm wide, hyaline to yellowish in KOH and yellowish to yellow in Melzer’s reagent. Clamp connections absent in all tissues.

##### Habitat and distribution.

Gregarious on soil in rainforest; currently known in Australia.

##### Notes.

*Abtylopilus australiensis* is characterized by its reddish-brown pileus; white context staining dark red at first and then becoming black when injured; reddish to orange-red hymenophore staining dark red initially and then blackish when bruised; orange-red, brownish red to blackish stipe; smooth basidiospores; and trichoderm pileipellis. Phylogenetically, *Ab. australiensis* clusters with *Ab. scabrosus* and forms a sister group with *Ab. indonesiensis*, and they are morphologically similar to each other. However, *Ab. scabrosus* differs from *Ab. australiensis* in its grayish red to brownish red pileus covered with tomentose squamules; gray to grayish pink hymenophore; white to dingy white stipe covered with dark scabrous squamules; and relatively large basidia measuring 28–55 × 16–17 μm (Li and Yang 2021). *Abtylopilus indonesiensis* differs from *Ab. australiensis* in its chocolate-brown to black pileus, pinkish hymenophore, pinkish orange stipe that is deep purple to black toward the base, and relatively small basidia measuring 26–40 × 10–11 μm (Li and Yang 2021).

#### ﻿ Abtylopilus indonesiensis

Taxon classificationAnimaliaBoletalesBoletaceae

Yan C. Li, J. Li, Halling, Osmundson & Zhu L. Yang sp. nov.

352825CD-FAC5-5FDD-98D8-3CF7C2CDCD0B

859186

Figs 7, 8h

##### Etymology.

*indonesiensis* referring to the species being found in Indonesia.

##### Type.

INDONESIA • Java, Haurbentes Park, alt. ca 300 m, 6.5442°S, 106.438°E, 16 Jan 2001, Halling 8070 (Holotype: BO; Isotype: NY 1393740).

##### Diagnosis.

*Abtylopilus indonesiensis* differs from other species of *Abtylopilus* in its a chocolate-brown to black pileus becoming a pale pinkish brown with age, a pinkish orange stipe but deep purple to black downward, a pinkish hymenophore usually, a whitish context changing to orange-red at first and then black when injured, and trichoderm pileipellis composed of 1.5–3.5 μm wide vertically arranged hyphae.

##### Description.

Basidiomata medium to large sized. Pileus 4–10 cm in diameter, convex to plano-convex, subvelutinous to tomentose, dry, chocolate-brown (7C3-7C4) to black (4F6), fading to a pale pinkish brown; context 1–1.5 cm thick, whitish, changing to orange-red then blackish when injured. Hymenophore adnate to adnexed; surface pinkish (7A2-7A3), staining orange-red at first and then black when bruised; tubes up to 8 mm long, white initially then pinkish to flesh colored when matured, staining orange-red at first and then black when bruised; pores angular, concolorous with hymenophoral surface and staining likewise. Stipe 4–6 × 1.5–2 cm, equal, dry, subpruinose, white to pinkish orange (7A3-7B3) at apex, deep purple (13E5-13E7) to black (4F6) downwards, staining red initially then blackish when injured. Odor and taste mild.

**Figure 7.**
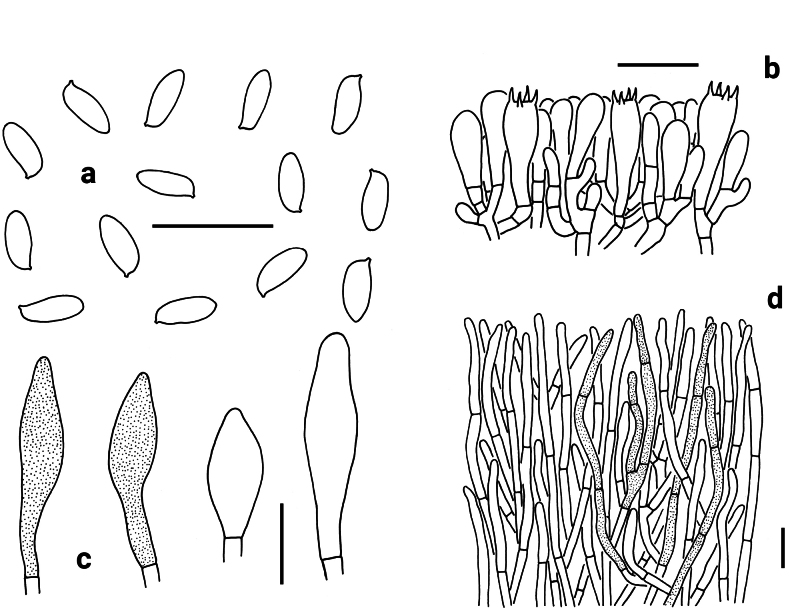
Microscopic features of *Abtylopilus indonesiensis* (NY 1393740, type). **a** Basidiospores **b** Basidia **c** Cheilo- and pleurocystidia **d** Pileipellis. Scale bars: 20 μm.

Basidia 26–40 × 10–11 μm, clavate, thin-walled, 4-spored, hyaline to yellowish in KOH. Basidiospores [60/3/1] 9.0–10.5 × 4–5 μm [Q = (1.90) 2.00–2.71 (2.86), Q_m_ = 2.31 ± 0.22], subfusiform in profile view with slight suprahilar depression, elongated to fusiform in ventral view, smooth, slightly thick-walled (up to 0.5 μm), hyaline to yellowish in KOH, yellowish to yellowish brown in Melzer’s reagent. Hymenophoral trama boletoid; hyphae cylindrical, hyaline to yellowish in KOH, yellowish in Melzer’s reagent. Cheilocystidia 32–55 × 11–14 μm, fusiform or subfusiform, thin-walled, brownish to yellowish brown in KOH, yellow to yellow-brown in Melzer’s reagent. Pleurocystidia morphologically similar to cheilocystidia. Pileipellis a palisadoderm, composed of 1.5–3.5 μm wide vertically arranged hyphae, yellowish brown to brownish in KOH and yellow-brown to dark brown in Melzer’s reagent; terminal cells 15–35 × 2–3 μm, subfusiform to cystidioid, thin-walled. Pileal trama composed of thin-walled hyphae; hyphae 2–3 μm wide, hyaline to yellowish in KOH and yellowish to yellow in Melzer’s reagent. Clamp connections absent in all tissues.

**Figure 8.**
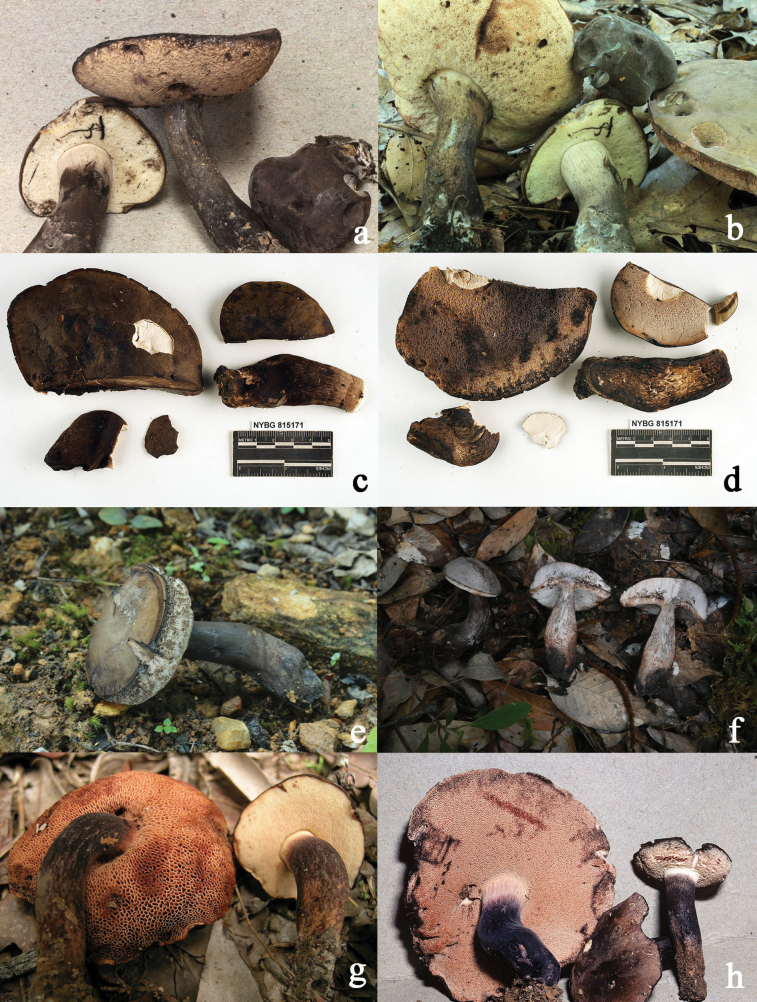
Basidiomata of “*Tylopilus alboater*” from different regions. **a, b***N. alboater* from North America (NY 1193926, images by Halling) **c, d***N. atronicotianus* from North America (NY 815171, images by Both) **e, f***Neoporphyrellus sinoalboater* from China (**e**KUN-HKAS 145312, type; **f**KUN-HKAS 145313) **g***Abtylopilus australiensis* from Australia (NY 2049854, image by Osmundson) **h***Ab. indonesiensis* from Indonesia (NY 1393740, image by Halling) (c–h from https://sweetgum.nybg.org/science/vh/specimen-list/).

##### Habitat and distribution.

Gregarious on the ground of dipterocarp forest (*Shorea*, *Dipterocarpus*, *Hopea*); currently known from Indonesia.

##### Notes.

*Abtylopilus indonesiensis* is characterized by a chocolate-brown to black pileus becoming pale pinkish brown with age; a pinkish orange stipe that is deep purple to black toward the base, staining reddish brown at first and then becoming blackish when bruised; a pinkish hymenophore usually staining reddish and then blackish when bruised; a whitish context changing to orange red at first and then black when injured; smooth basidiospores; and trichoderm pileipellis. *Abtylopilus indonesiensis* is phylogenetically related to *Ab. scabrosus* and *Ab. australiensis*. However, *Ab. scabrosus* differs from *Ab. indonesiensis* in its grayish red to brownish red or ruby pileus, white to dingy white stipe densely covered with dark scabrous squamules, and relatively large basidia measuring 28–55 × 16–17 μm (Li and Yang 2021). *Abtylopilus australiensis* differs from *Ab. indonesiensis* in its reddish-brown pileus, reddish to orange-red hymenophore, and reddish brown to blackish stipe densely minute-pruinose (Li and Yang 2021).

## ﻿Discussion

According to our morphological studies and phylogenetic analyses, specimens from North America, Asia, and Australia labeled as “*Tylopilus alboater*” represent eight species belonging to four known genera—*Abtylopilus*, *Anthracoporus*, *Indoporus*, and *Tylopilus*—and one new genus, *Neoporphyrellus*. Specimens from East and Southeast Asia represent four species, including two known species, viz. *I. squamulosus* and *An. cystidiatus*, and two new species, *N. sinoalboater* and *Ab. indonesiensis*. Specimens from America represent three species, including two new combinations, viz. *N. alboater* and *N. atronicotianus*, and one known species, viz. *T. rubrobrunneus*. A specimen from Australia represents a new species of *Abtylopilus*, viz., *Ab. australiensis*.

Phylogenetically, the new genus *Neoporphyrellus* is nested within the *Boletoideae* and clusters as a sister group to *Afroboletus*, *Strobilomyces*, *Indoporus*, and *Brasilioporus*. These genera can be distinguished from each other based on morphological and molecular features. So far, the true *N. alboater* has only been reported from the eastern USA, occurring in forests dominated by *Quercus* (Bessette et al. 2000, 2024). *Neoporphyrellus sinoalboater* is currently known from central and southwest China, where it is found in forests dominated by *Quercus semicarpifolia*. *Neoporphyrellus atronicotianus*, originally described by Both (1998) based on specimens from New York and known only from the eastern USA, occurs in mixed forests dominated by *Quercus*, *Tsuga*, and *Fagus* (Bessette et al. 2000, 2024). Morphologically, the three species of *Neoporphyrellus* can be distinguished from each other by the colors of the pileus, stipe, and hymenophore; the sizes of the hymenophoral pores and tubes; the shapes of the cheilocystidia and pleurocystidia; and the size of the basidia.

Species of *Abtylopilus* were first reported from China, but according to our results, species of this genus also occur in Indonesia and Australia. Therefore, it is likely that many species have not yet been scientifically described or remain to be discovered. Similarly, more species of *Neoporphyrellus* may be discovered in the future, which will further clarify species diversity and distribution.

## Supplementary Material

XML Treatment for Neoporphyrellus

XML Treatment for Neoporphyrellus alboater

XML Treatment for Neoporphyrellus atronicotianus

XML Treatment for Neoporphyrellus sinoalboater

XML Treatment for Abtylopilus

XML Treatment for Abtylopilus australiensis

XML Treatment for Abtylopilus indonesiensis

## ﻿Additional information

### Use of AI

No use of AI was reported.

### Adherence to national and international regulations

All the fungal strains used in this study have been legally obtained, respecting the Convention on Biological Diversity (Rio Convention).

### Author ORCIDs

Jin Li https://orcid.org/0000-0001-8377-9184

Roy E. Halling https://orcid.org/0000-0002-2610-3062

Todd W. Osmundson https://orcid.org/0000-0002-0551-5228

Zhu L. Yang https://orcid.org/0000-0001-9745-8453

Yan-Chun Li https://orcid.org/0000-0002-7109-9269

### Data availability

Sequences generated in this study are available in the NCBI GenBank database under the accessions provided in Table 1. The matrix used for phylogenetic analyses has been submitted to TreeBASE.

## Supplementary materials

Supplementary material 1 Assembly accession, species name, voucher, BUSCO Assessment Results of genomes used in phylogenomic analyses Data type xlsx https://binary.pensoft.net/file/1455235This dataset is made available under the Open Database License (http://opendatacommons.org/licenses/odbl/1.0/). The Open Database License (ODbL) is a license agreement intended to allow users to freely share, modify, and use this Dataset while maintaining this same freedom for others, provided that the original source and author(s) are credited.Jin Li, Roy E. Halling, Todd W. Osmundson, Zhu L. Yang, Yan-Chun Li

Supplementary material 2 The results of AU-test, including value of bp-RELL, p-KH, p-SH, c-ELW, p-AU Data type xlsx https://binary.pensoft.net/file/1455236This dataset is made available under the Open Database License (http://opendatacommons.org/licenses/odbl/1.0/). The Open Database License (ODbL) is a license agreement intended to allow users to freely share, modify, and use this Dataset while maintaining this same freedom for others, provided that the original source and author(s) are credited.Jin Li, Roy E. Halling, Todd W. Osmundson, Zhu L. Yang, Yan-Chun Li

Supplementary material 3 Best model Data type nex Explanation note The bset model of each partition in phylogenetic analysis and phylogenomic analysis seleting by automaticlly selecting in ModelFinder. https://binary.pensoft.net/file/1455237This dataset is made available under the Open Database License (http://opendatacommons.org/licenses/odbl/1.0/). The Open Database License (ODbL) is a license agreement intended to allow users to freely share, modify, and use this Dataset while maintaining this same freedom for others, provided that the original source and author(s) are credited.Jin Li, Roy E. Halling, Todd W. Osmundson, Zhu L. Yang, Yan-Chun Li

Supplementary material 4 Bayes.log Data type txt Explanation note The log file of MrBayes, the ESS values are listed here. https://binary.pensoft.net/file/1455238This dataset is made available under the Open Database License (http://opendatacommons.org/licenses/odbl/1.0/). The Open Database License (ODbL) is a license agreement intended to allow users to freely share, modify, and use this Dataset while maintaining this same freedom for others, provided that the original source and author(s) are credited.Jin Li, Roy E. Halling, Todd W. Osmundson, Zhu L. Yang, Yan-Chun Li

Supplementary material 5 *Boletoideae* lsu, *tef1*, *rpb1*, *rpb2* Data type fas Explanation note The combined dataset (*nrLSU* + *rpb1* + *rpb2* + *tef1-α*) used in ML and MB phylogenetic analyses including 100 sequences (representing 23 genera of *Boletoideae* and two genus of *Suillelloideae*) consisted of 3056 nucleotides. https://binary.pensoft.net/file/1455239This dataset is made available under the Open Database License (http://opendatacommons.org/licenses/odbl/1.0/). The Open Database License (ODbL) is a license agreement intended to allow users to freely share, modify, and use this Dataset while maintaining this same freedom for others, provided that the original source and author(s) are credited.Jin Li, Roy E. Halling, Todd W. Osmundson, Zhu L. Yang, Yan-Chun Li

Supplementary material 6 nrLSU gene trees Data type jpg Explanation note The tree of the nuclear ribosomal large subunit (nrLSU). https://binary.pensoft.net/file/1455240This dataset is made available under the Open Database License (http://opendatacommons.org/licenses/odbl/1.0/). The Open Database License (ODbL) is a license agreement intended to allow users to freely share, modify, and use this Dataset while maintaining this same freedom for others, provided that the original source and author(s) are credited.Jin Li, Roy E. Halling, Todd W. Osmundson, Zhu L. Yang, Yan-Chun Li

Supplementary material 7 tef1-α gene tree Data type jpg Explanation note The tree of the translation elongation factor 1-α gene (tef1-α). https://binary.pensoft.net/file/1455241This dataset is made available under the Open Database License (http://opendatacommons.org/licenses/odbl/1.0/). The Open Database License (ODbL) is a license agreement intended to allow users to freely share, modify, and use this Dataset while maintaining this same freedom for others, provided that the original source and author(s) are credited.Jin Li, Roy E. Halling, Todd W. Osmundson, Zhu L. Yang, Yan-Chun Li

Supplementary material 8 rpb1 gene tree Data type jpg Explanation note The tree of the largest subunit of RNA polymerase II (rpb1). https://binary.pensoft.net/file/1455242This dataset is made available under the Open Database License (http://opendatacommons.org/licenses/odbl/1.0/). The Open Database License (ODbL) is a license agreement intended to allow users to freely share, modify, and use this Dataset while maintaining this same freedom for others, provided that the original source and author(s) are credited.Jin Li, Roy E. Halling, Todd W. Osmundson, Zhu L. Yang, Yan-Chun Li

Supplementary material 9 rpb2 gene tree Data type jpg Explanation note The tree of the second-largest subunit of RNA polymerase II (rpb2). https://binary.pensoft.net/file/1455243This dataset is made available under the Open Database License (http://opendatacommons.org/licenses/odbl/1.0/). The Open Database License (ODbL) is a license agreement intended to allow users to freely share, modify, and use this Dataset while maintaining this same freedom for others, provided that the original source and author(s) are credited.Jin Li, Roy E. Halling, Todd W. Osmundson, Zhu L. Yang, Yan-Chun Li
