# Supplementary figures and images for: ﻿Global diversity of the Tylopilus alboater complex (Boletaceae, Boletales): new genus and species, and typification of the name Boletus alboater
Source: IMA Fungus. 2025 Oct 31;16:e159676. doi: 10.3897/imafungus.16.159676 (PMC12595509; doi:10.3897/imafungus.16.159676)

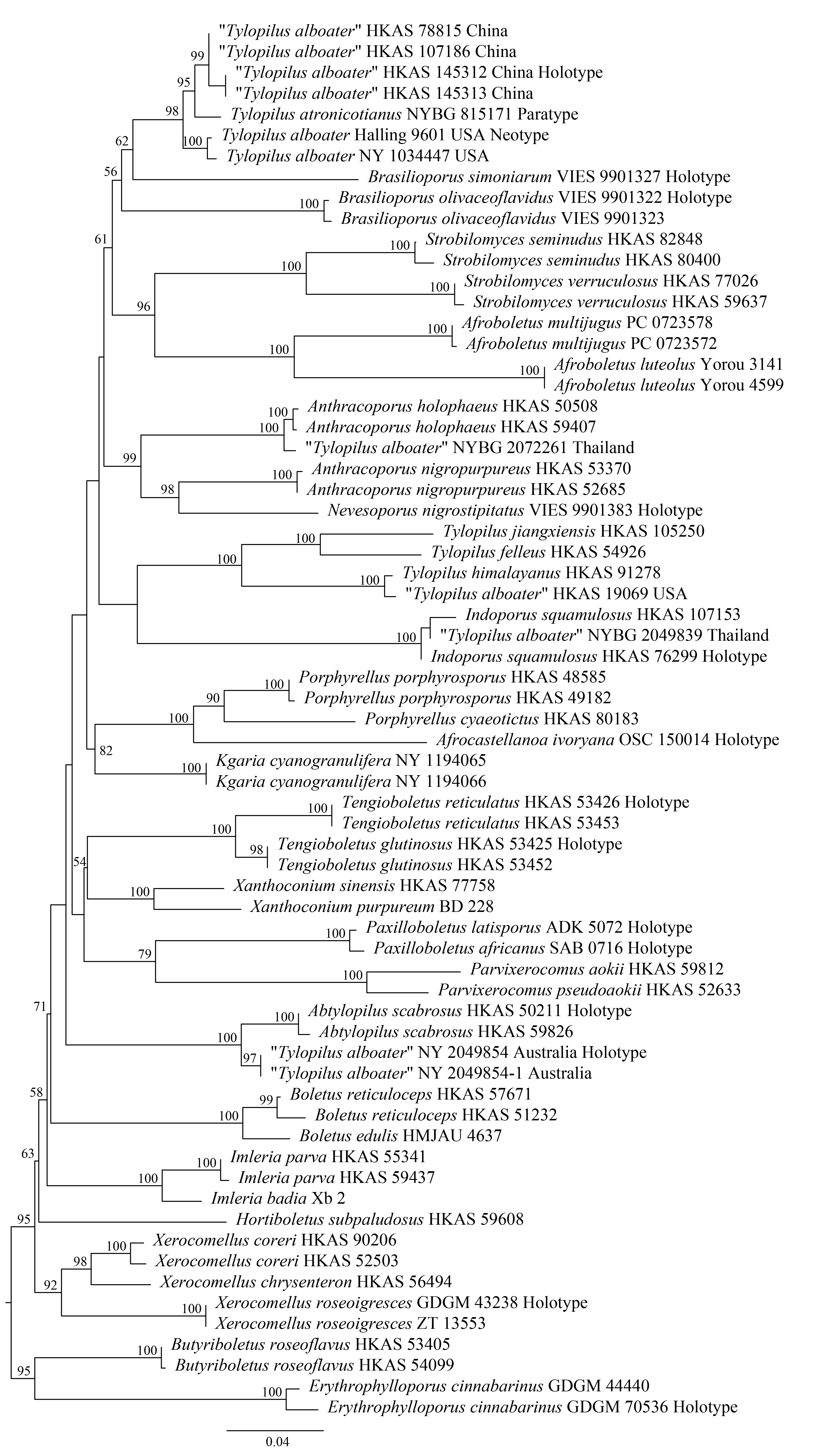

Supplement: Supplementary material 7 — tef1-α gene tree [file imafungus-16-e159676-s007.jpg]

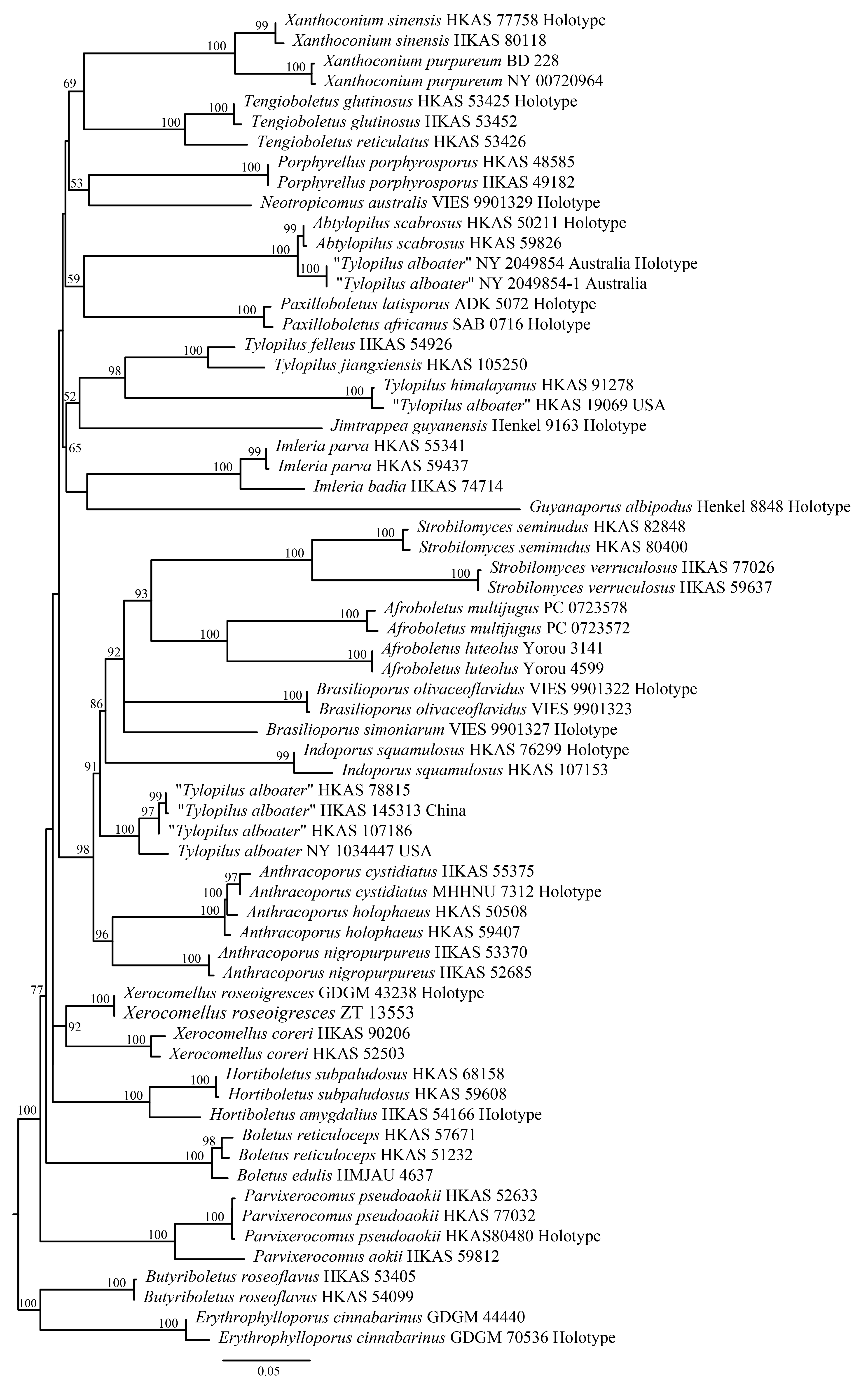

Supplement: Supplementary material 8 — rpb1 gene tree [file imafungus-16-e159676-s008.jpg]

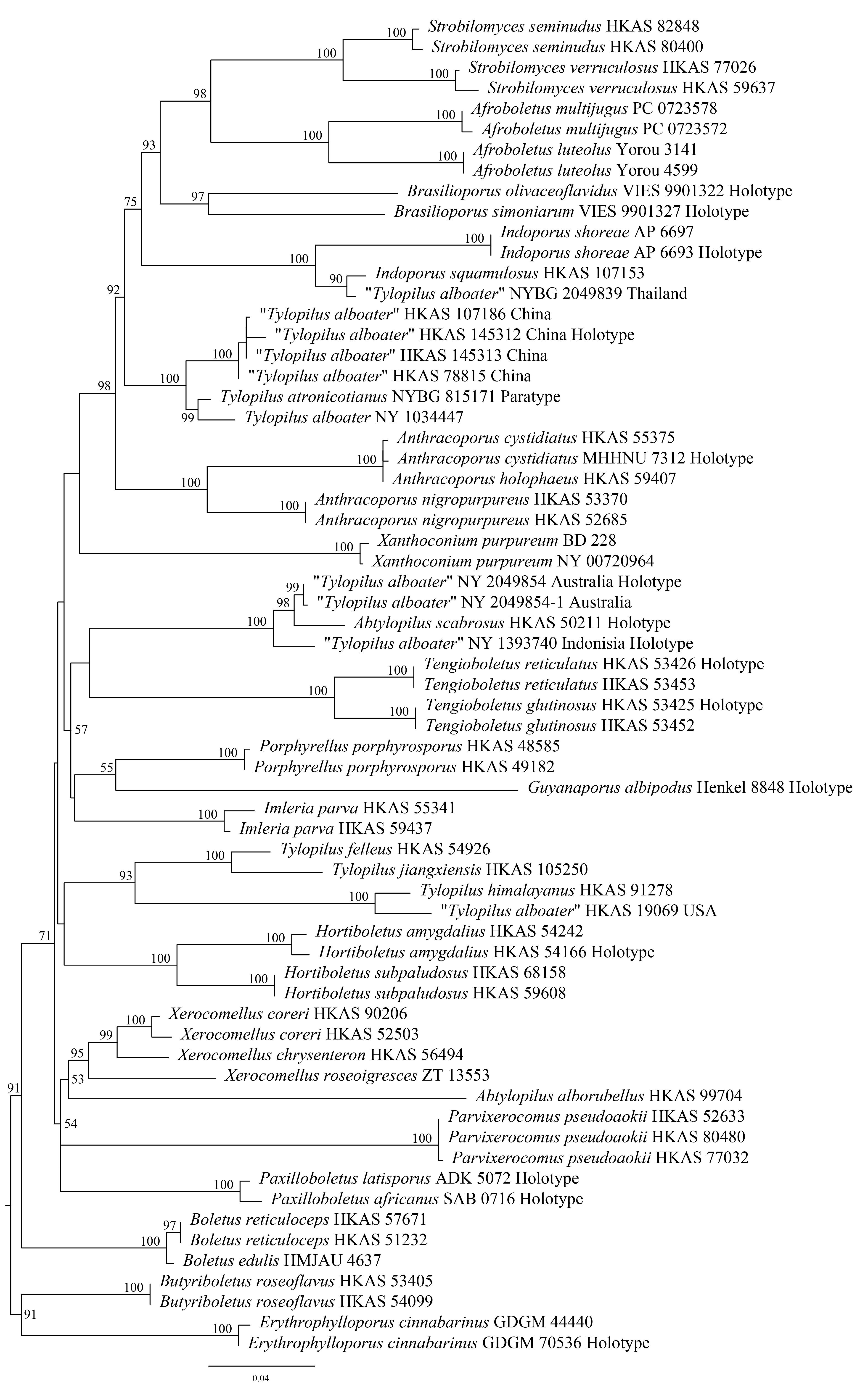

Supplement: Supplementary material 9 — rpb2 gene tree [file imafungus-16-e159676-s009.jpg]
